# Formation of ER-lumenal intermediates during export of *Plasmodium* proteins containing transmembrane-like hydrophobic sequences

**DOI:** 10.1371/journal.ppat.1011281

**Published:** 2023-03-31

**Authors:** Yvette S. Levray, Bianca Bana, Sarah J. Tarr, Emilia J. McLaughlin, Peter Rossi-Smith, Anita Waltho, Georgina H. Charlton, Riccardo Zenezini Chiozzi, Colin R. Straton, Konstantinos Thalassinos, Andrew R. Osborne

**Affiliations:** 1 Institute of Structural and Molecular Biology, University College London, London, United Kingdom; 2 Institute of Structural and Molecular Biology, Department of Biological Sciences, Birkbeck, London, United Kingdom; 3 Department of Infectious Disease, Faculty of Medicine, Imperial College London, London, United Kingdom; 4 UCL Mass Spectrometry Science Technology Platform, Division of Biosciences, University College London, London, United Kingdom; Johns Hopkins University, UNITED STATES

## Abstract

During the blood stage of a malaria infection, malaria parasites export both soluble and membrane proteins into the erythrocytes in which they reside. Exported proteins are trafficked via the parasite endoplasmic reticulum and secretory pathway, before being exported across the parasitophorous vacuole membrane into the erythrocyte. Transport across the parasitophorous vacuole membrane requires protein unfolding, and in the case of membrane proteins, extraction from the parasite plasma membrane. We show that trafficking of the exported *Plasmodium* protein, Pf332, differs from that of canonical eukaryotic soluble-secreted and transmembrane proteins. Pf332 is initially ER-targeted by an internal hydrophobic sequence that unlike a signal peptide, is not proteolytically removed, and unlike a transmembrane segment, does not span the ER membrane. Rather, both termini of the hydrophobic sequence enter the ER lumen and the ER-lumenal species is a productive intermediate for protein export. Furthermore, we show in intact cells, that two other exported membrane proteins, SBP1 and MAHRP2, assume a lumenal topology within the parasite secretory pathway. Although the addition of a C-terminal ER-retention sequence, recognised by the lumenal domain of the KDEL receptor, does not completely block export of SBP1 and MAHRP2, it does enhance their retention in the parasite ER. This indicates that a sub-population of each protein adopts an ER-lumenal state that is an intermediate in the export process. Overall, this suggests that although many exported proteins traverse the parasite secretory pathway as typical soluble or membrane proteins, some exported proteins that are ER-targeted by a transmembrane segment-like, internal, non-cleaved hydrophobic segment, do not integrate into the ER membrane, and form an ER-lumenal species that is a productive export intermediate. This represents a novel means, not seen in typical membrane proteins found in model systems, by which exported transmembrane-like proteins can be targeted and trafficked within the lumen of the secretory pathway.

## Author summary

Symptoms of malaria occur during the blood stage of infection when the malaria parasite resides inside human red blood cells. Hundreds of proteins, synthesized by the parasite, are exported into the host cell where they modify its properties. Some exported proteins become embedded in membranes and are referred to as membrane proteins. Despite their importance in disease pathology, how these membrane proteins are transported into the red blood cell is poorly understood. Some exported membrane proteins are thought to become membrane-embedded in the parasite endoplasmic reticulum and are subsequently extracted from the parasite plasma membrane before being transported into the red blood cell where they are then re-inserted into the appropriate membrane. Contrary to this, we find that a subset of membrane proteins enter into the lumen of the endoplasmic reticulum and either do not insert into the parasite endoplasmic reticulum membrane or insert and are rapidly extracted. This behaviour is very different from the behaviour of non-exported membrane proteins that have been studied in model systems such as human or yeast cells, and suggests that the trafficking of exported membrane proteins is a mechanistically distinct process that may represent a unique drug target.

## Introduction

Within the infected red blood cell, the malaria parasite resides inside a membrane compartment, the parasitophorous vacuole. The parasite exports hundreds of proteins across the parasitophorous vacuole membrane and into the red blood cell, where they play key roles in disease pathogenesis, nutrient uptake, modification of the erythrocyte cytoskeleton, cytoadhesion, and immune evasion [1–6]. Two classes of proteins are exported into the red blood cell: proteins that contain a PEXEL (*Plasmodium* export element) sequence and proteins that lack a PEXEL sequence (PEXEL-negative exported proteins or PNEPs) [7–11]. For PEXEL-containing proteins, cleavage of the PEXEL by the protease plasmepsin V in the endoplasmic reticulum (ER) lumen is necessary for export [10–14].

Within the red blood cell, some exported proteins reside in the cytoplasm as soluble proteins, while others integrate into membrane structures known as Maurer’s clefts or into the red blood cell plasma membrane. In the case of exported soluble proteins, after targeting to the parasite ER, proteins are thought to progress down the secretory pathway by vesicle transport before being released into the parasitophorous vacuole [15]. Translocation across the parasitophorous vacuole membrane is mediated by the PTEX complex (*Plasmodium* translocon of exported proteins), which comprises multiple proteins including an HSP101 ATPase, PTEX150, PTEX88 and a pore forming protein, EXP2 [16,17]. The PTEX complex unfolds these exported proteins and feeds them through the EXP2 pore in the parasitophorous vacuole membrane [16–20]. Additionally, the Exported protein-interacting complex (EPIC) is also localised in the parasitophorous vacuole and facilitates PTEX-mediated protein export [21]. Recent experiments suggest that HSP101 initially associates with soluble exported proteins in the parasite ER and then escorts them to the parasitophorous vacuole. Binding of HSP101 to EXP2 and other PTEX proteins then reconstitutes the PTEX complex at the parasitophorous vacuole membrane [22]. Export of membrane proteins is more complex and several different mechanisms have been proposed in which membrane proteins are extracted from a parasite membrane. Some exported membrane proteins, including REX2, are thought to integrate into the parasite ER membrane before being trafficked through the secretory pathway, extracted from the parasite plasma membrane, and translocated across the parasitophorous vacuole membrane via the PTEX complex [23,24]. An alternative model has been proposed for trafficking of the exported membrane protein PfEMP1. Subcellular fractionation studies support a model in which PfEMP1 can form a non-membrane-integrated species within the parasite ER and parasitophorous vacuole [21,25]. However, both exported soluble and exported membrane proteins converge on the PTEX complex in the parasitophorous vacuole [24], and components of this complex are important for parasite survival and virulence [18,19,26–28].

Understanding the initial step of protein export within the parasite endoplasmic reticulum is essential to our understanding of the overall export process. In eukaryotes, the majority of proteins destined for the secretory pathway are targeted to the ER via the Sec61 channel that can facilitate co- and post-translational translocation. In the latter pathway, a complex of Sec62-63 and the lumenal chaperone BiP associate with the Sec61 channel [29]. Significantly, in *Plasmodium*, a distinct Sec61 complex that includes SPC25, Sec62 and plasmepsin V, is responsible for translocation of a subset of PEXEL-containing proteins [30].

In eukaryotes, most soluble proteins that are translocated into the ER lumen are targeted to the Sec61 channel by an N-terminal signal peptide. Typically, a signal peptide comprises a short stretch of 6–12 hydrophobic residues close to the protein N-terminus. Signal peptides do not integrate stably into the ER membrane and as they are cleaved co-translationally, are not present in mature proteins (cleavage is typically mediated by signal peptidase but in the case of many PEXEL-containing parasite proteins, plasmepsin V fulfils this role [30]). Eukaryotic membrane proteins can be targeted to the Sec61 channel by a signal peptide, but in the absence of a signal peptide, targeting is mediated by the first transmembrane segment. Transmembrane segments are typically more hydrophobic than signal peptides, containing 18 or more hydrophobic residues. Importantly, the Sec61 channel mediates translocation of signal peptide-containing soluble proteins across the ER membrane, but also facilitates the integration of transmembrane segments into the ER membrane. The channel can therefore open in two directions: perpendicular to the plane of the membrane to allow translocation of polypeptides across the membrane, and laterally to allow exit of transmembrane segments into the lipid bilayer [31,32]. As polypeptides traverse the Sec61 channel they sample the hydrophilic interior of the channel and the hydrophobic lipid environment in the lateral exit of the channel. Hydrophobic polypeptide segments have a propensity to partition into the hydrophobic bilayer and thus integrate into the membrane, whereas soluble proteins have a propensity to remain within the hydrophilic centre of the channel and thus pass into the ER lumen. The ‘choice’ between ER membrane translocation or integration is dictated by the hydrophobicity of the particular polypeptide sequence [33].

These principles appear to be universally conserved in both the eukaryotic Sec61 channel and its prokaryotic homologue the SecY channel [33–35]. Indeed, in model systems most Sec61-targeted membrane proteins rapidly assume a membrane-integrated, sodium carbonate insoluble state. Consequently, many exported parasite proteins that appear to contain an internal, non-cleaved hydrophobic sequence that resembles a transmembrane segment, would be expected to partition into the ER membrane during translocation through the Sec61 channel, and form a monotopic membrane protein rather than a soluble lumenal protein. Consistent with this, exported membrane proteins including REX2, STEVOR and RIFIN are integrated in the ER membrane [23,36–39]. However, when trapped in the parasite ER by Brefeldin A treatment, PfEMP1 proteins predominantly form a sodium carbonate soluble species [25]. Whilst one study showed that SBP1 is sodium carbonate insoluble in Brefeldin A treated parasites [40], others have found SBP1 to be partially sodium carbonate extractable after Brefeldin A treatment [41]. Similarly, whilst the exported parasite protein Pf332 is not thought to integrate into a membrane within the red blood cell [40], analysis of its sequence suggests that in the context of ER-targeting Pf332 resembles a membrane protein: it does not contain a signal peptide but does contain an internal hydrophobic segment between residues 546 and 568, which resembles a transmembrane segment [42]. However, if Pf332 is trapped within the parasite ER using Brefeldin A, only a minor proportion of the protein is found in the membrane fraction [40]. This suggests that for these proteins a fraction of the population may traffic through the parasite secretory pathway in a non-membrane-integrated state. However, it is known that some proteins with less hydrophobic transmembrane segments, embedded in membranes with a high protein-lipid ratio, may be extracted with sodium carbonate [25,43]. Additionally, membrane proteins that fail to fold correctly and are destined for degradation can also assume a soluble non-membrane-integrated state [44].

Consequently, it remains unclear whether some PEXEL-negative exported proteins are extracted from the ER membrane and trafficked as soluble lumenal species to the parasitophorous vacuole, or whether they are integrated into the ER membrane and subsequently extracted from the parasite plasma membrane. In our current experiments, we analyse the export of Pf332, SBP1 and MAHRP2, all of which are targeted to the ER by hydrophobic sequences that in the context of ER-targeting resemble transmembrane segments. We show that export of these proteins is completely or partially blocked by the addition of a C-terminal ER-retention sequence, indicating that the productive export species for these proteins have their C-termini within the ER lumen. Circumventing the possible problem that exported proteins may behave anomalously upon sodium carbonate extraction and subcellular fractionation, we use split-GFP in live cells, to determine whether the ER-trapped proteins reside in the ER membrane or have translocated entirely into the ER lumen. Analysis of Pf332 and SBP1 proteins trapped in the ER, either by an ER retention sequence or using Brefeldin A, shows that both their N- and C-termini enter the ER lumen and that the proteins form an entirely ER lumenal species. Additionally, the C-terminus of MAHRP2 also enters the ER-lumen. These data indicate that for a subset of PEXEL-negative exported proteins that resemble single-spanning membrane proteins, either the entire protein population or a fraction of the population, enters the ER lumen and that the lumenal species is a productive intermediate in the export pathway into the red blood cell.

## Results

### The N-terminal sequence and putative transmembrane segment of Pf332 are important for protein export

**Fig 1:**
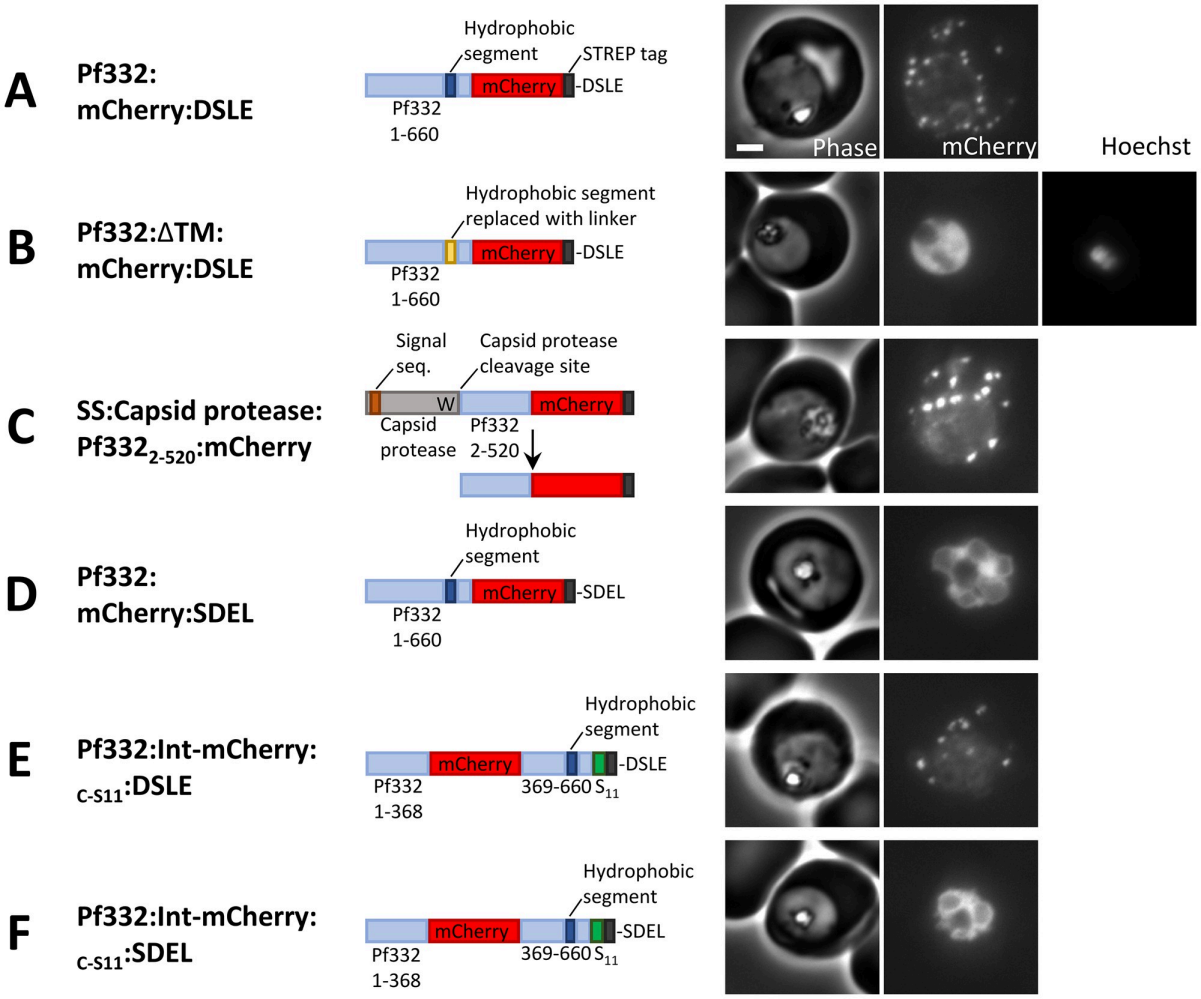
Export of a Pf332 model protein into the infected red blood cell. (A-F) Phase contrast and fluorescence images of parasites expressing the indicated proteins. Scale bar: 2 μm.

The Maurer’s cleft protein Pf332 is 6093 residues in length and contains a putative transmembrane segment between residues 546 and 568 [45]. The predicted ΔG for this transmembrane segment is -2.218 (this value is indicative of the hydrophobicity and indicates the propensity to partition into a bilayer [33,37]. See S1 Table for comparison to other proteins). To characterise the trafficking of Pf332, an mCherry-tagged model protein comprising residues 1–660 was assembled. The protein construct Pf332:mCherry:DSLE includes the putative transmembrane segment, 92 residues following the transmembrane segment, and a C-terminal mCherry tag, followed by a STREP tag (Fig 1A). The final four residues in this construct correspond to the sequence DSLE; the significance of these residues will be apparent in later experiments. When expressed in parasites, the protein was exported into the red blood cell and localised in punctae that colocalised with the Maurer’s cleft marker MAHRP1 (Figs 1A and S1A lane 2 and S2A).

Pf332 is not predicted to contain a signal peptide suggesting that the putative transmembrane segment mediates ER-targeting. Consistent with this, replacement of the putative transmembrane segment with a short hydrophilic linker, corresponding to the sequence RRASTSAGRSS, leads to accumulation of the protein in the parasite cytoplasm (Figs 1B and S1A, lane 5).

To determine whether the N-terminal sequence of Pf332 that precedes the putative transmembrane domain contains sufficient information to mediate translocation across the parasitophorous vacuole membrane (as seen in other PNEPs and cleaved PEXEL proteins [23,46]), a fusion of this protein fragment with a viral self-cleaving capsid protease was assembled [46]. The capsid protease cleaves itself after a C-terminal tryptophan residue [47]. The capsid protease domain was preceded by a signal peptide and followed by residues 2–520 of Pf332 and mCherry (this fragment ends before the putative transmembrane segment of Pf332). Self-cleavage of the protease should release a protein fragment, comprising Pf332 residues 2–520 with a C-terminal mCherry tag, within the ER lumen (Fig 1C). The first methionine of Pf332 was omitted from the construct as this residue would normally be removed from endogenous Pf332 by methionine amino peptidase prior to targeting to the parasite ER. The protein was expressed in parasites and efficient protease cleavage was confirmed by western blotting (S1A Fig, lane 8). The mCherry-tagged Pf332 protein fragment was efficiently exported into the red blood cell cytoplasm and localised in punctate structures that colocalise with MAHRP1 (Figs 1C and S2B). Together, these data indicate that the hydrophobic segment between residues 546 and 568 mediates ER-targeting of Pf332 and that the N-terminal residues 2–520 contain an export sequence that is sufficient to mediate translocation across the parasitophorous vacuole membrane and targeting to the Maurer’s clefts.

### A productive intermediate in the export of Pf332 has both N- and C-termini within the ER lumen

To test whether Pf332 is trafficked from the ER as a lumenal species, a C-terminal ER retention sequence (SDEL) was added to the protein [36,38,48,49]; Pf332:mCherry:SDEL accumulated in the parasite (Figs 1D and S1A lane 3 and S1B lane 2) colocalizing with the ER-marker plasmepsin V (S2C Fig). The protein Pf332:mCherry:DSLE is identical except that the C-terminal residues correspond to the sequence DSLE, a scrambled version of the SDEL sequence, and is exported into the red blood cell, as mentioned above (Fig 1A). Given that the KDEL receptor recognises proteins within the lumen of the secretory pathway [50,51], this indicates that the C-terminus of Pf332 is within the ER lumen and that a species with its C-terminus in the ER lumen is a productive intermediate in the export pathway.

To test whether the position of the mCherry tag influenced the behaviour of the protein, we performed similar experiments in which the mCherry tag was inserted internally between residues 368 and 369 of Pf332, on the N-terminal side of the putative transmembrane segment; a split-GFP S11 tag was also added at the C-terminal end of the protein (Figs 1E, 1F and S1B, lane 3,4). The protein with the scrambled DSLE sequence (Pf332:Int-mCherry:_C-S11_:DSLE) was exported into the red blood cell (Figs 1E and S2D) but the equivalent protein with a C-terminal SDEL sequence (Pf332:Int-mCherry:_C-S11_:SDEL) was retained within the parasite (Fig 1F). This indicates that Pf332 retention by an SDEL sequence is efficient regardless of the position of the mCherry tag.

**Fig 2:**
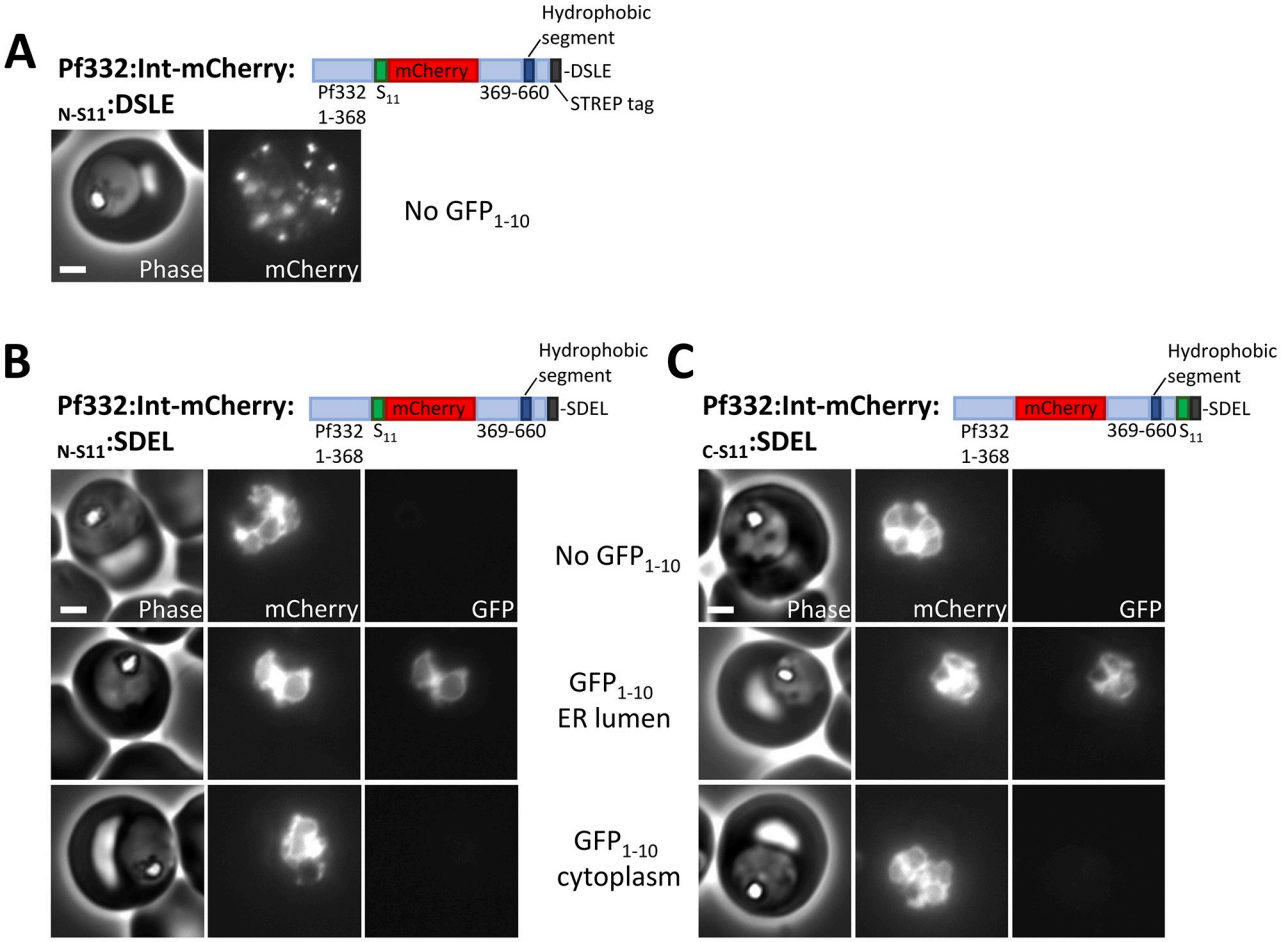
ER-lumenal localisation of ER-retained Pf332. (A-C) Phase contrast and fluorescence images of parasites expressing the indicated Pf332 proteins either alone or with the indicated GFP_1-10_ proteins. Scale bar: 2 μm.

To test whether both N- and C-terminal ends of Pf332 are ER-lumenal, and hence whether the putative transmembrane segment has translocated into the ER lumen, split-GFP was used. GFP can be split into two separate non-fluorescent polypeptides comprising ß-strands 1–10 or ß-strand 11, referred to as GFP_1-10_ and S11, respectively [52]. The GFP_1-10_ and S11-tag used in these experiments do not require fusion to interacting proteins in order to interact but can only reconstitute a fluorescent protein complex if they both reside within the same cellular compartment [53,54]. Tagging of Pf332 with the S11 sequence and co-expression with GFP_1-10_ fragments in the parasite cytoplasm or ER was used to determine the topology of the protein. The Pf332 protein described above with a C-terminal S11 tag was used, Pf332:Int-mCherry:_C-S11_:SDEL (Fig 1F). Two additional constructs were also assembled in which the S11 tag was placed prior to the mCherry sequence on the N-terminal side of the putative transmembrane segment; Pf332:Int-mCherry:_N-S11_:DSLE and Pf332:Int-mCherry:_N-S11_:SDEL (Figs 2A, 2B and S1C). As expected, Pf332 proteins with either N- or C-terminal S11 tags and the C-terminal sequence DSLE were exported into the red blood cell (Figs 1E and 2A), and the proteins with the C-terminal SDEL sequences were retained within the parasite (Figs 1F, 2B and 2C).

The ER-retained Pf332 proteins were then co-expressed with cytoplasmic GFP_1-10_ or GFP_1-10_ targeted to the parasite ER lumen by a signal peptide and retained by a C-terminal SDEL sequence (referred to as ER-lumenal GFP_1-10_). GFP_1-10_ expression cassettes were integrated into the *pfs47* gene locus using Cas9 as this allows robust protein expression in the majority of parasites (S1 Text). GFP fluorescence was reconstituted when Pf332:Int-mCherry:_N-S11_:SDEL was co-expressed with ER-lumenal GFP_1-10_ but not when co-expressed with cytoplasmic GFP_1-10_ (Fig 2B). Similarly, GFP fluorescence was reconstituted only when Pf332:Int-mCherry:_C-S11_:SDEL was co-expressed with ER-lumenal GFP_1-10_ but not cytoplasmic GFP_1-10_ (Fig 2C). Immunofluorescence labelling of parasites expressing only ER-lumenal GFP_1-10_ with an anti-GFP antibody, confirmed its ER localisation as it colocalised with the ER-marker plasmepsin V (S2E Fig). Expression of both GFP_1-10_ fragments was confirmed by western blotting (S1C Fig). Although cytoplasmic GFP_1-10_ is consistently present at lower levels than the ER-lumenal GFP_1-10_, it is functional and expressed at sufficient levels to reconstitute GFP fluorescence when co-expressed with plasmepsin V that has a cytoplasmic C-terminal S11 tag (S3A and S3B Figs) or when co-expressed with cytoplasmic S11-tagged mCherry (S3C and S3D Figs).

Given that interaction of GFP_1-10_ and an S11-tagged protein is essential for the split-GFP method to work, it is likely that expression of ER-lumenal GFP_1-10_, that itself has a C-terminal SDEL sequence, may lead to some retention of a protein that passes through the ER lumen. Indeed, although we see robust export of Pf332:Int-mCherry:_C-S11_:DSLE into punctae within the infected red blood cell when co-expressed with ER-lumenal GFP_1-10_, the GFP_1-10_ protein does lead to increased retention of the protein within the parasite (S2F Fig). Although it would be preferable that expression of split-GFP fragments had no influence on trafficking of a protein passing through the ER, use of split-GFP remains the only method that can be used to determine protein topology in live intact parasites in which cell integrity has not been disrupted. Most importantly, all Pf332 proteins tested were efficiently retained in the parasite ER by addition of an SDEL sequence, regardless of whether they were expressed with an ER-lumenal GFP_1-10_ protein.

Taken together, these experiments show that the hydrophobic segment of Pf332 targets the protein to the parasite ER, but unlike conventional ER proteins, rather than integrating into the ER membrane, both N- and C-terminal ends of the hydrophobic segment translocate into the ER lumen. This ER-lumenal protein is a productive intermediate in the export of this protein as addition of the C-terminal SDEL sequence inhibits export.

### Translocation of the Pf332 hydrophobic segment into the ER lumen

To provide further support for a model in which the hydrophobic segment in Pf332 can enter the ER lumen, we made a series of exported protein constructs containing the Pf332 hydrophobic segment, whose behaviour could only be explained if both N- and C-terminal ends of the Pf332 hydrophobic segment have a propensity to be translocated into the ER lumen. Significantly, the following experiments are not reliant on exogenously expressed split-GFP but rely on the activity of the endogenous proteins, KDEL receptor and plasmepsin V, to infer the topology and functional significance of a particular topology.

**Fig 3:**
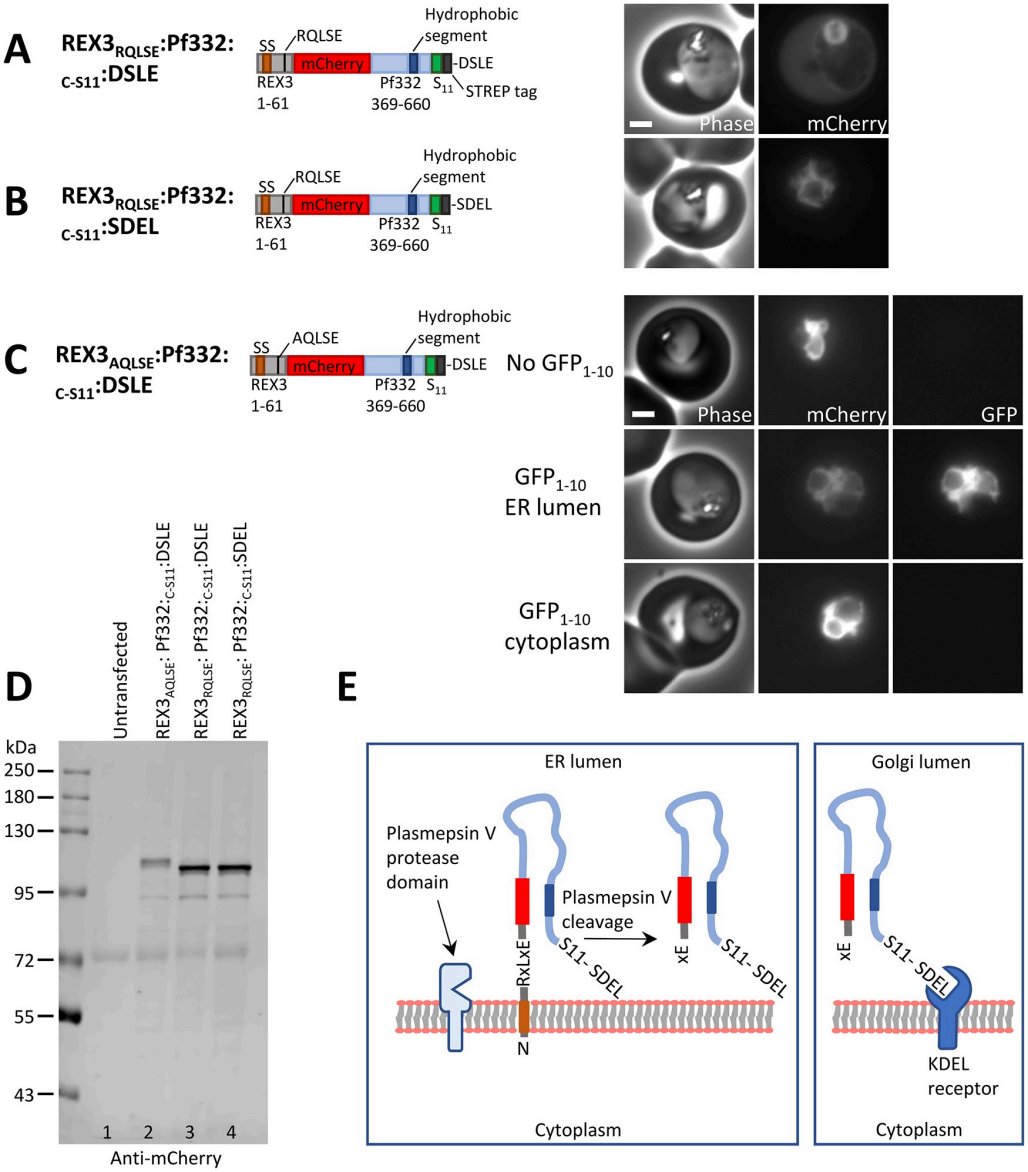
The putative TM segment of Pf332 translocates into the ER lumen. (A-B) Phase contrast and fluorescence images of parasites expressing the indicated proteins. (C) Phase and fluorescence images of parasites expressing REX3_AQLSE_:Pf332:_C-S11_:DSLE alone, or co-expressing either ER-lumenal GFP_1-10_ or cytoplasmic GFP_1-10_, as indicated. Scale bar: 2 μm. (D) Western blot of parasites expressing the indicated proteins (probed with an anti-mCherry antibody). (E) Cartoon representation of plasmepsin V cleavage of the PEXEL sequence in REX3_RQLSE_:Pf332:_C-S11_:SDEL within the ER and recognition of the C-terminal SDEL sequence in the Golgi lumen.

To do this, the construct REX3_RQLSE_:Pf332:_C-S11_:DSLE was assembled (Fig 3A). This comprises the N-terminal 61 residues of REX3, fused to mCherry, and followed by residues 369–660 of Pf332, which includes the putative Pf332 transmembrane segment. At the C-terminus of Pf332 an S11 tag, followed by the sequence DSLE, was added. REX3 is a PEXEL-containing exported soluble protein and its N-terminal 61 residues include a signal peptide/transmembrane domain followed by a PEXEL sequence (RQLSE), that are sufficient to target a protein for export into the red blood cell [9,55]. The protease domain of plasmepsin V is within the ER lumen and cleaves the PEXEL of REX3 between the leucine and serine residue [46,54].

The REX3 portion of the model protein REX3_RQLSE_:Pf332:_C-S11_:DSLE is expected to place the N-terminal end of the fusion protein in the ER lumen, where the PEXEL can be cleaved by plasmepsin V (Fig 3E). Plasmepsin V cleavage of the PEXEL would therefore be indicative of this portion of the polypeptide being within the ER lumen. REX3_RQLSE_:Pf332:_C-S11_:DSLE and a similar model protein REX3_AQLSE_:Pf332:_C-S11_:DSLE, in which the PEXEL sequence is mutated to AQLSE, were expressed in parasites. Western blotting indicated that REX3_RQLSE_:Pf332:_C-S11_:DSLE migrates with an approximate molecular mass of 110 kDa (Fig 3D, lane 3). Mutation of the PEXEL sequence to AQLSE, which should prevent cleavage by plasmepsin V [12,14], led to an increase in the molecular weight of the protein (Fig 3D; compare lanes 2 and 3). These data indicate that the PEXEL is cleaved by plasmepsin V, and therefore the N-terminal end of the Pf332 hydrophobic segment of this protein is located within the ER lumen.

REX3_RQLSE_:Pf332:_C-S11_:DSLE is exported and is diffusely localised in the red blood cell cytoplasm (Fig 3A). Mutation of the PEXEL sequence (REX3_AQLSE_:Pf332:_C-S11_:DSLE), results in the protein being retained in the parasite (Fig 3C), and addition of a C-terminal SDEL sequence (REX3_RQLSE_:Pf332:_C-S11_:SDEL) also leads to retention of the protein within the parasite (Fig 3B). Given that REX3_RQLSE_:Pf332:_C-S11_:SDEL is retained within the parasite, this indicates that the C-terminus of the protein is in the ER lumen (Fig 3E). To determine the location of the N-terminal end of this ER-retained protein, it was purified for tryptic digestion and analysed by mass spectrometry. The most N-terminal peptides retrieved corresponded to N-acetylated and non-acetylated SEPVVEEQDLK and SEPVVEEQDLKK. These peptides correspond to the expected N-terminal sequence following PEXEL cleavage by plasmepsin V, indicating that the N-terminus of this protein is also in the ER lumen. These data indicate that both N- and C-terminal ends of REX3_RQLSE_:Pf332:_C-S11_:SDEL are located within the ER lumen and consequently that in the context of this protein, the putative transmembrane segment of Pf332 has a propensity to translocate into the ER lumen rather than integrate into the ER membrane.

Additionally, when REX3_AQLSE_:Pf332:_C-S11_:DSLE was co-expressed with ER-lumenal GFP_1-10_, but not cytoplasmic GFP_1-10_, green fluorescence was observed (Figs 3C and S4A), confirming that the C-terminus of this protein is located in the ER lumen. This further supports the model in which the Pf332 hydrophobic segment either does not integrate into the membrane during translocation into the ER or is efficiently extracted from the ER membrane. Consistent with the model and previous data [40], Pf332:mCherry:DSLE and the ER-retained Pf332:mCherry:SDEL are predominantly sodium carbonate soluble (S4B Fig).

Taken together, the data in the preceding sections demonstrate the existence of an export pathway in which the protein Pf332 can be targeted to the ER lumen by an internal hydrophobic segment. Critically, in this pathway, the ER-targeting sequence does not resemble a canonical signal peptide (it is not at the N-terminus and is not proteolytically removed). The behaviour of Pf332 also differs from that of a canonical transmembrane protein as ultimately the hydrophobic segment appears to translocate across the ER membrane rather than integrate into it.

### Topology of SBP1 trapped in the parasite ER after Brefeldin A treatment

To test whether other exported proteins can also be translocated into the lumen of the secretory pathway, the PEXEL-negative exported protein SBP1 was analysed. SBP1 contains a single transmembrane segment (predicted ΔG for this transmembrane segment is -3.599; S1 Table) that ultimately integrates into the Maurer’s cleft membrane [56]. Cell fractionation and protease protection experiments suggest SBP1 also undergoes membrane extraction during export but this is thought to occur at the parasite plasma membrane [23,24]. Analysis of SBP1 using sodium carbonate extraction, either in the presence or absence of Brefeldin A, has yielded contradictory results showing that SBP1 is either predominantly in the membrane fraction, or equally distributed in both the sodium carbonate soluble and insoluble fractions [36,41]. Consistent with the latter, we find that an mCherry tagged SBP1 is distributed in both the sodium carbonate soluble and membrane fractions (S4C Fig, lanes 1,2,3). Analysis of the tagged protein after Brefeldin A treatment is precluded by the very low expression levels after prolonged treatment necessary to completely retain the protein in the ER. In the following experiments we aimed to test whether the SBP1 can be translocated into a lumenal compartment of the secretory pathway; critically, these experiments aimed to test this hypothesis in intact cells.

**Fig 4:**
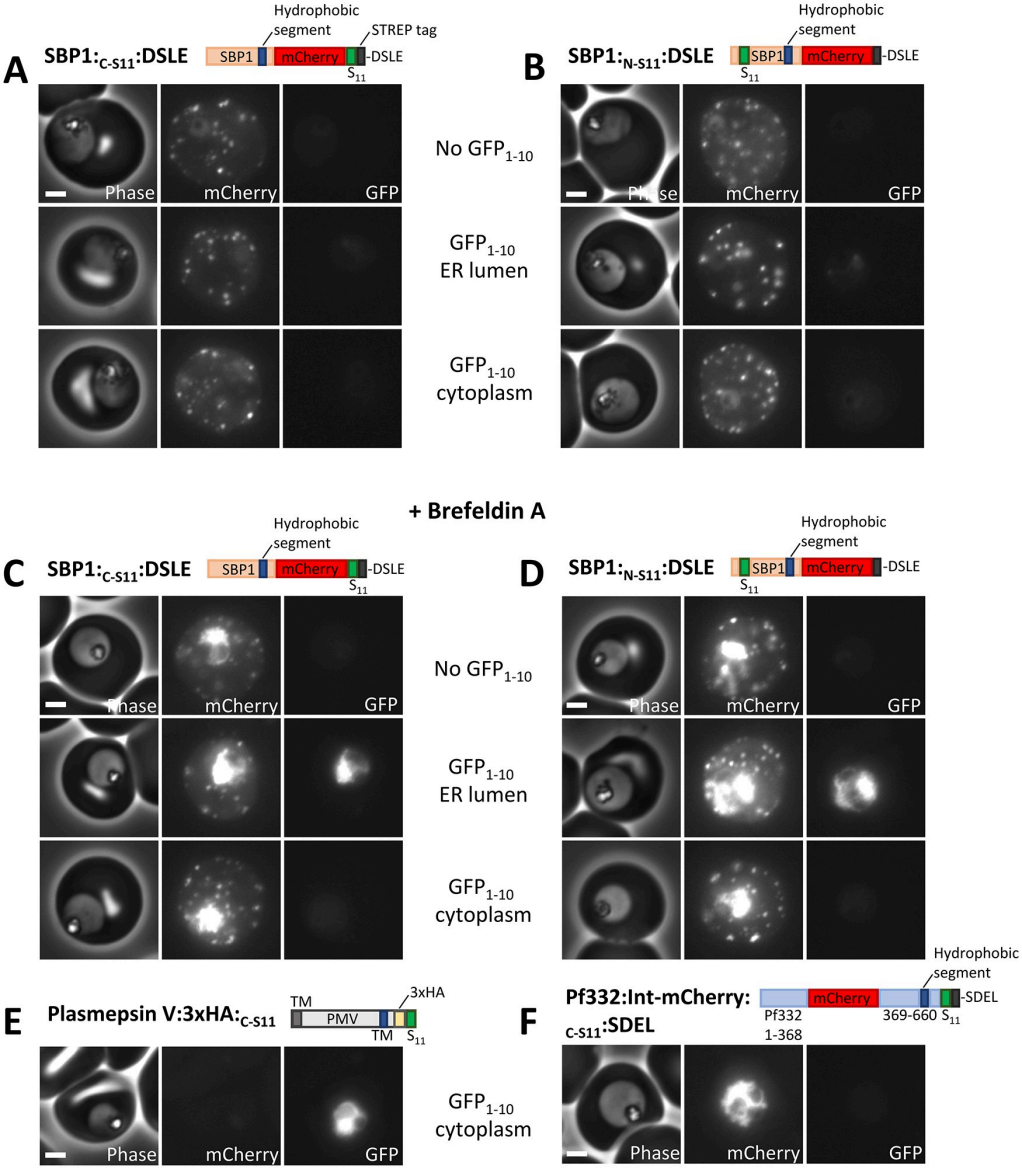
ER-lumenal location of SBP1 in Brefeldin A-treated parasites. (A-B) Phase contrast and fluorescence images of parasites expressing the indicated proteins are shown. Proteins were expressed alone, co-expressed with ER-lumenal GFP_1-10_ or cytoplasmic GFP_1-10_, as indicated. (C-F) Phase contrast and fluorescence images of Brefeldin A-treated parasites expressing the indicated proteins. Proteins were expressed alone, co-expressed with ER-lumenal GFP_1-10_ or cytoplasmic GFP_1-10_, as indicated. Parasites were treated with 1μg/ml Brefeldin A for four hours prior to imaging. Scale bar: 2 μm.

SBP1 with C-terminal mCherry and S11 tags, followed by the C-terminal residues DSLE (SBP1:_C-S11_:DSLE), or a similar construct (SBP1:_N-S11_:DSLE) in which the S11 tag is placed at the N-terminus between residues 22 and 23 of SBP1, were expressed in parasites. Both proteins were exported into the red blood cell with little accumulation of the SBP1 protein within the parasite (Figs 4A, 4B and S5A). Analysis of SBP1:_C-S11_:DSLE by immunofluorescence labelling showed that the exported protein co-localised with the Maurer’s cleft protein MAHRP1 (S5C Fig). When co-expressed with ER-lumenal or cytoplasmic GFP_1-10_, little GFP fluorescence was detectable for either protein (Figs 4A and 4B).

In order to trap SBP1 in the parasite ER, cultures were treated for four hours with 1μg/ml of Brefeldin A. While this does not completely block protein export, it is sufficient to cause detectable accumulation of SBP1 protein within the parasite (Figs 4C and 4D). The protein accumulated within the parasite colocalised with the ER-marker plasmepsin V (S5D Fig). Treatment with DMSO alone did not lead to ER-accumulation of SBP1 (S5E Fig). In Brefeldin A-treated parasites expressing SBP1:_C-S11_:DSLE, GFP fluorescence was robustly detectable in the presence of ER-lumenal GFP_1-10_ (Fig 4C). In the presence of cytoplasmic GFP_1-10_ only a very weak cytoplasmic fluorescence close to background level was detectable, but this was diffusely localised throughout the parasite and was not concentrated around the ER-trapped SBP1 (Fig 4C, see also S6 Fig for high contrast GFP images). Similarly, after Brefeldin A treatment of parasites expressing SBP1:_N-S11_:DSLE, GFP fluorescence was robustly detectable in the presence of ER-lumenal GFP_1-10_ and only a very weak diffuse GFP fluorescence close to background level was detectable in the presence of cytoplasmic GFP_1-10_ (Figs 4D and S6).

Although neither of the above proteins reconstituted a fluorescent GFP complex when expressed with cytoplasmic GFP_1-10_, this GFP_1-10_ fragment protein is functional after Brefeldin A treatment as GFP fluorescence was detectable in treated parasites co-expressing cytoplasmic GFP_1-10_ and plasmepsin V with a C-terminal, cytoplasmic S11 sequence (Fig 4E). Additionally, the integrity of the ER was not compromised, as no GFP fluorescence was seen in Brefeldin A-treated parasites co-expressing the ER-lumenal protein Pf332:Int-mCherry:_C-S11_:SDEL and cytoplasmic GFP_1-10_ (Fig 4F).

Taken together, these results indicate that both the N- and C-terminal ends of SBP1 reside in the ER lumen after the protein is trapped in the parasite ER using Brefeldin A. This is consistent with a model in which the protein is efficiently extracted into a lumenal compartment of the secretory pathway during the export process.

### Trafficking of SBP1 is partially altered by an ER-retention sequence

**Fig 5:**
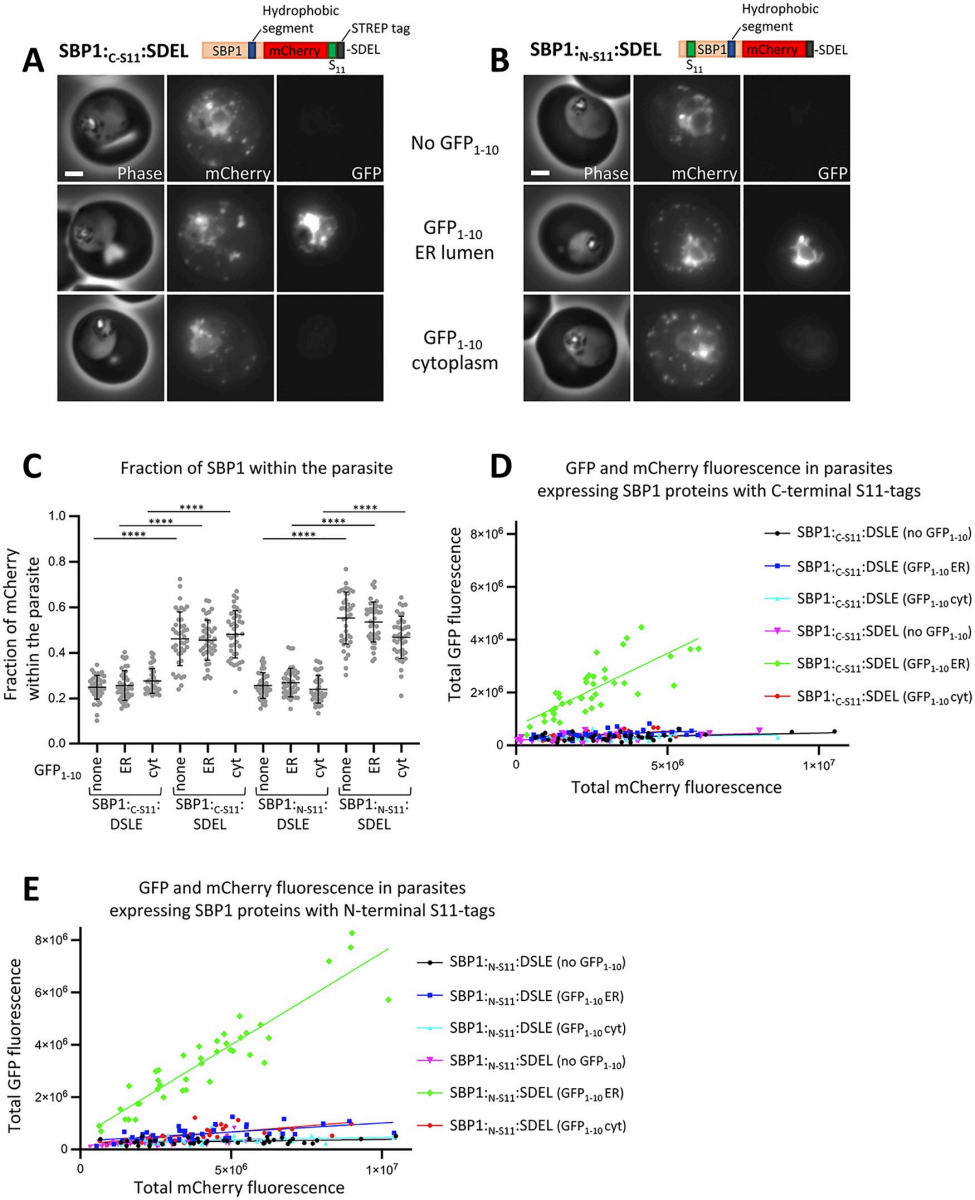
Export of SBP1 is perturbed by a C-terminal ER-retention sequence. (A-B) Phase contrast and fluorescence images of parasites expressing the indicated proteins are shown. Proteins were expressed alone, co-expressed with ER-lumenal GFP_1-10_ or cytoplasmic GFP_1-10_, as indicated. Scale bar: 2 μm. (C) The fraction of total mCherry fluorescence located within the parasite is shown for parasite lines expressing the indicated SBP1 and GFP_1-10_ proteins. Forty individual trophozoite stage parasites, from two independent experiments, were analysed for each parasite line. Data points for individual parasites, mean and standard deviation are shown. P-values were determined using a one-way ANOVA test, P < 0.0001 = ****. (D-E) For parasites expressing the indicated SBP1 proteins, the total mCherry fluorescence and total GFP fluorescence levels are plotted (for both channels this corresponds to the fluorescence in the infected red blood cell and the parasite). Forty individual trophozoite stage parasites, from two independent experiments, were analysed for each parasite line.

To test whether an ER-lumenal species is a productive intermediate in the export of SBP1, we tested whether an SDEL sequence can influence its trafficking. If SBP1 efficiently integrates into the parasite ER membrane an SDEL sequence should have no impact on protein export. Only if the C-terminus of the protein is lumenal should an SDEL sequence affect export. As discussed above, the SBP1 proteins with a DSLE C-terminal sequence (SBP1:_C-S11_:DSLE or SBP1:_N-S11_:DSLE) were efficiently exported into the red blood cell (Figs 4A and 4B). Equivalent proteins with an SDEL ER-retention sequence (SBP1:_C-S11_:SDEL or SBP1:_N-S11_:SDEL) were also exported into the red blood cell but some protein accumulation in the parasite was apparent (Figs 5A, 5B and S5A). Quantitative analysis shows that SBP1 mCherry fluorescence distribution is variable between individual parasites, but that an SDEL sequence causes an increase in the proportion of parasite-retained SBP1-fluorescence in the majority of cells (Fig 5C).

To more robustly quantify the extent of ER retention and to further characterise retained SBP1, the proteins were co-expressed with ER-lumenal or cytoplasmic GFP_1-10_ proteins. When SBP1 proteins with C-terminal residues DSLE were expressed with either cytoplasmic or ER-lumenal GFP_1-10_, very little GFP fluorescence was detected indicating that the SBP1 proteins are exported and are not sufficiently abundant within the parasite cytoplasm or ER lumen to efficiently reconstitute GFP (Figs 4A, 4B, 5D and 5E; see S6A and S6B Figs for high contrast GFP images). However, when SBP1:_C-S11_:SDEL or SBP1:_N-S11_:SDEL are expressed with ER-lumenal GFP_1-10_, a clear GFP signal is observed within the parasite (Figs 5A, 5B, 5D and 5E). This indicates that although addition of an SDEL sequence does not completely block export, it increases the fraction of the protein trapped in the ER. Significantly, GFP is efficiently reconstituted in the presence of ER-lumenal GFP_1-10_ when the S11 tag is either on the N- or C-terminal side of the SBP1 transmembrane segment. Thus, for the fraction of SBP1 that is retained in the parasite ER by an SDEL sequence, the predominantly detected species has both N- and C-termini within the ER lumen.

Efficient reconstitution of GFP fluorescence is not seen when SBP1:_C-S11_:SDEL is co-expressed with cytoplasmic GFP_1-10_ (Fig 5A). In some parasites a very weak ER-like GFP signal is observed when SBP1:_N-S11_:SDEL is co-expressed with cytoplasmic GFP_1-10_ suggesting that a minor population of this protein construct may assume a topology with the N-terminus in the cytoplasm (See S6F Fig for high contrast GFP images). The significance of this minor population is unclear.

Overall these data indicate that SBP1 export is partially affected by addition of an SDEL sequence and that the predominately detected ER-retained species has both N- and C- termini within the ER lumen. This suggests that for at least a fraction of SBP1 protein an ER-lumenal species is a productive export intermediate.

### Topology of MAHRP2 trapped in the parasite ER after Brefeldin A treatment

**Fig 6:**
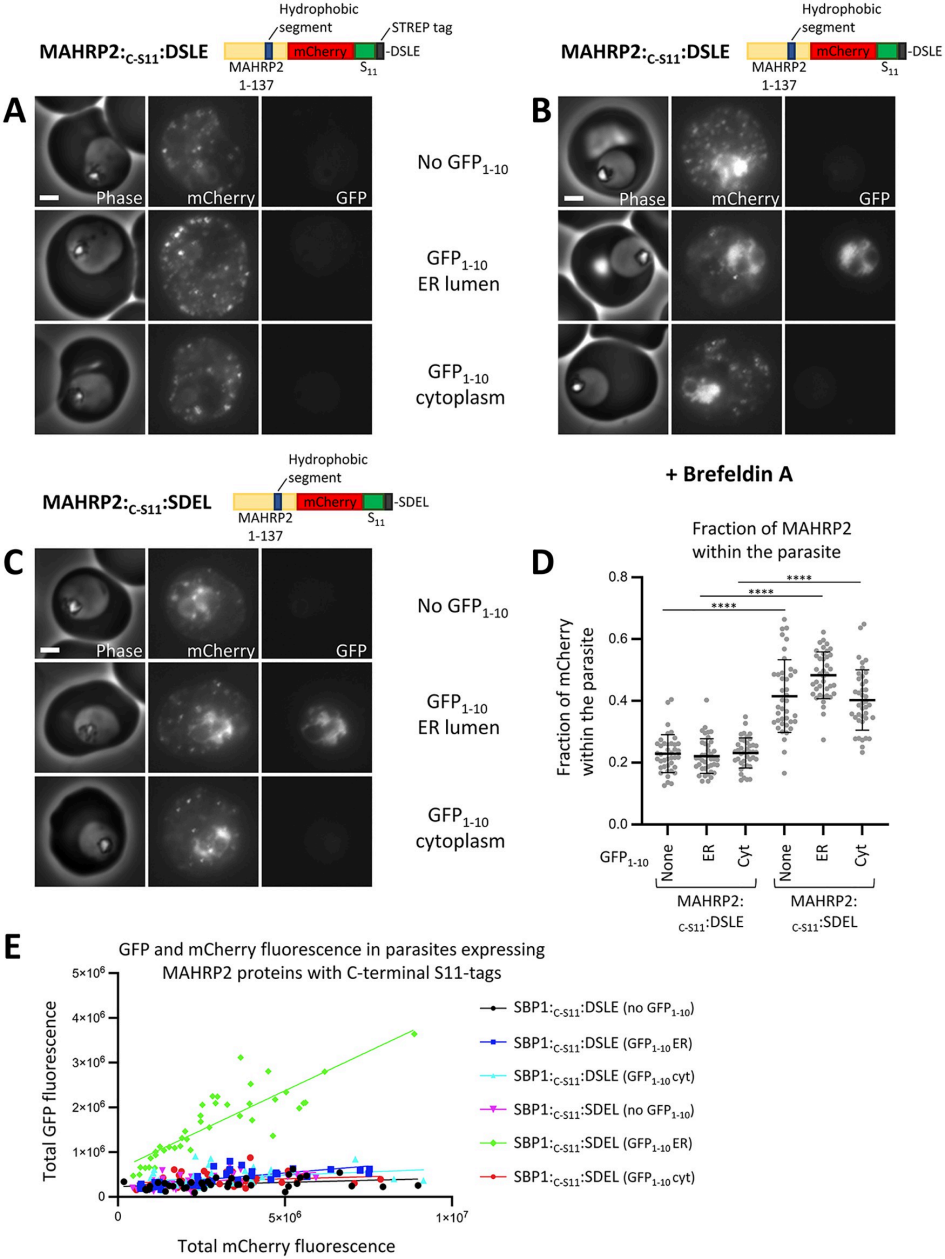
ER-lumenal location of the C-terminus of MAHRP2. (A) Phase contrast and fluorescence images of parasites expressing the indicated proteins are shown. Proteins were expressed alone, co-expressed with ER-lumenal GFP_1-10_ or cytoplasmic GFP_1-10_, as indicated. (B) Images of parasites expressing MAHRP2:_C-S11_:DSLE expressed alone, co-expressed with ER-lumenal GFP_1-10_ or cytoplasmic GFP_1-10_, as indicated. Parasites were treated with 1μg/ml Brefeldin A for four hours prior to imaging. (C) Images of parasites expressing MAHRP2:_C-S11_:SDEL and the indicated GFP_1-10_ proteins, are shown. Scale bar: 2 μm. (D) The fraction of total mCherry fluorescence located within the parasite is shown for each parasite line expressing the indicated MAHRP2 and GFP_1-10_ proteins. Forty individual trophozoite stage parasites, from four independent experiments, were analysed for each parasite line. Data points for individual parasites, mean, and standard deviation are shown. P-values were determined using a one-way ANOVA test, P < 0.0001 = ****. (E) For parasites expressing the indicated MAHRP2 and GFP_1-10_ proteins, the total mCherry fluorescence and total GFP fluorescence levels are plotted. Forty individual trophozoite stage parasites, from four independent experiments, were analysed for each parasite line.

Having established that at least a fraction of SBP1 protein can enter the ER lumen, we aimed to test whether another exported protein behaves similarly. MAHRP2 is a PEXEL-negative exported protein that is peripherally membrane associated in the red blood cell and localises to Maurer’s clefts tether structures [57]. Previous studies find MAHRP2 to be predominantly sodium carbonate soluble [57]. In sodium carbonate extraction experiments, we find a significant proportion of the tagged MAHRP2 is within the membrane fraction but a significant fraction is also sodium carbonate soluble (S4C Fig, lanes 4,5,6). Like Pf332 and SBP1, MAHRP2 is targeted to the ER via a non-cleaved, internal hydrophobic segment that in the context of ER-targeting resembles a transmembrane domain [57]. The predicted ΔG for this transmembrane segment is -3.679 (S1 Table). Insertion, deletion or mutation of sequences within the N-terminus of MAHRP2 abolish export, indicating that this region is critical for protein export and cannot be altered. However, a tag can be added to the C-terminus of the protein without disrupting export [57]. Therefore, to determine whether the C-terminus of MAHRP2 resides in the parasite cytoplasm or is translocated into a lumenal compartment of the secretory pathway, a C-terminal mCherry tag followed by an S11 tag was added to the protein (MAHRP2:_C-S11_:DSLE) (Fig 6A).

MAHRP2:_C-S11_:DSLE was efficiently exported into the red blood cell with little mCherry fluorescence retained within the parasite (Figs 6A, S7A and S7B) and the exported protein mostly colocalises or is adjacent to the Maurer’s cleft marker MAHRP1 (S7C Fig) as shown previously [57]. When co-expressed with ER-lumenal GFP_1-10_, the MAHRP2:_C-S11_:DSLE protein was robustly exported and only very low green signal was detected (Fig 6A). When expressed in the presence of cytoplasmic GFP_1-10_, MAHRP2:_C-S11_:DSLE was efficiently exported (Fig 6A). Very little GFP signal was visible when co-expressed with cytoplasmic GFP_1-10_; the low level of green fluorescence in these parasites was diffusely localised in the parasite cytoplasm (Fig 6A; see S8 Fig for high contrast GFP images).

To further analyse the topology of MAHRP2:_C-S11_:DSLE within the parasite, cultures were treated with Brefeldin A. After addition of Brefeldin A (but not DMSO), accumulation of red fluorescence was readily detectable within the parasite ER (Figs 6B, S7D and S7E). Brefeldin A treatment of parasites co-expressing GFP_1-10_ fragments indicated that the predominant species of MAHRP2:_C-S11_:DSLE that is trapped within the parasite has its C-terminus within the ER lumen. Only very weak and diffuse cytoplasmic green fluorescence was observed in the presence of cytoplasmic GFP_1-10_ (Fig 6B; see S8 Fig for high contrast GFP images).

### Trafficking of MAHRP2 is perturbed by an ER-retention sequence

To determine whether MAHRP2 trafficking is altered by addition of an SDEL sequence, parasites expressing MAHRP2:_C-S11_:DSLE or MAHRP2:_C-S11_:SDEL were compared. In both cases protein export was observed but the fraction of red fluorescence retained within the parasite was increased by the addition of a C-terminal SDEL sequence (Figs 6C and 6D). When MAHRP2:_C-S11_:SDEL was co-expressed with ER-lumenal GFP_1-10_, a robust ER-like GFP fluorescence was observed (Figs 6C and 6E). Conversely, when co-expressed with cytoplasmic GFP_1-10_ only a weak diffuse green fluorescence localised in the cytoplasm was observed (Figs 6C and 6E). Quantification of the total GFP-derived fluorescence of parasites expressing MAHRP2 constructs indicates that a minor fraction of the protein that ends with the DSLE sequence resides in the ER with its C-terminus within the ER lumen. However, the level of green fluorescence is enhanced by the addition of an SDEL sequence indicating that the SDEL sequence increases ER-retention and partially perturbs the trafficking of the protein (Fig 6E).

Taken together, these results suggest that during trafficking into the red blood cell, MAHRP2 can assume a topology in which the C-terminus resides within a lumenal compartment of the secretory pathway. As addition of a C-terminal SDEL sequence increases the retention of the protein within the parasite, this suggests that a species in which the C-terminus is ER-lumenal is a productive export intermediate for at least a fraction of MAHRP2 protein. Notably, after either Brefeldin A treatment or addition of an SDEL sequence, an ER-trapped species in which the C-terminus of the protein was localised in the parasite cytoplasm, was not robustly detected.

## Discussion

Pf332, SBP1 and MAHRP2 are all targeted to the ER by an internal, non-cleaved hydrophobic polypeptide segment. Our current experiments, consistent with those of others [40], suggest that Pf332 is targeted to the parasite ER by an internal transmembrane domain-like hydrophobic sequence that does not integrate into the ER membrane. This represents a novel targeting mechanism that differs from the targeting of typical soluble or membrane proteins observed in model systems. Pf332 likely traffics through the parasite secretory pathway as a non-membrane-integrated lumenal species that is a productive intermediate in the export pathway.

The topology of SBP1 and MAHRP2 trapped in the ER with Brefeldin A, strongly supports a model in which these two proteins enter a lumenal compartment of the secretory pathway during export. Indeed, extraction of membrane proteins from the parasite ER membrane would be consistent with recent observations that HSP101 is partially ER-localised and can associate with exported proteins in the parasite ER [22]. Although our Brefeldin A experiments are consistent with an ER-extraction model, it is difficult to completely exclude extraction of these proteins occurring later in the secretory pathway as some non-ER resident proteins (which could include a novel protein translocase) may accumulate in the ER after Brefeldin A treatment. To reduce this problem, prolonged Brefeldin A treatments were avoided and parasites were Brefeldin A-treated for the shortest time that allowed detection of ER-accumulated exported proteins. Nonetheless, these observations support conclusions of previous fractionation experiments and provide the first evidence in live cells showing translocation of these proteins into a lumenal compartment [24,41]. The partial perturbation of the trafficking of these two proteins by an ER retention sequence further supports an ER membrane extraction model for at least a fraction of these proteins. Indeed, it is difficult to reconcile this observation with a membrane extraction model for these particular proteins, in which extraction from a parasite membrane and translocation across the parasitophorous vacuole membrane are tightly coupled as proposed for REX2 [24]. The simplest interpretation of our experiments would be that at least a fraction of each of these proteins forms an export-competent lumenal species within the parasite ER-recycling compartment.

The lack of extensive N-linked glycosylation has made it difficult to define the topology of proteins in the parasite. The use of split-GFP represents a partial solution to this problem [53,54]. In all experiments we were able to detect lumenal species of exported proteins but were unable to detect ER-localised species of Pf332, SBP1 or MAHRP2 in which the C-termini were cytoplasmic. Split-GFP and reconstitution of GFP fluorescence allows us to make a positive assignment of the predominantly detectable topology for a particular protein. Although both cytoplasmic and ER-lumenal GFP_1-10_ are functional, it is more difficult to demonstrate based on the lack of GFP fluorescence, that a particular topology is not present. This may be due to lower detection sensitivity (particularly with cytoplasmic GFP_1-10_ which is consistently present at a lower level), the transient nature of a particular topology, or an inability to form a GFP complex in a particular cellular context. Additionally, the ER-lumenal GFP_1-10_ can also contribute to the ER-retention of Pf332 that itself lacks an SDEL sequence. In the experiments using SBP1 and MAHRP1, ER-retention mediated by GFP_1-10_ is less apparent but as there is a fairly broad distribution of the data points, it is difficult to rule out some effect. However, in the experiments in which we use ER-lumenal GFP_1-10_ to quantify ER-retention of SBP1 and MAHRP1 proteins ending in DSLE or SDEL, any contribution of the GFP_1-10_ to ER retention should be similar in both parasite lines i.e. the difference seen should be due to the SDEL on the exported protein itself. Other methods will be required to globally determine protein topology of parasite exported proteins as they traffic through the secretory pathway.

**Fig 7:**
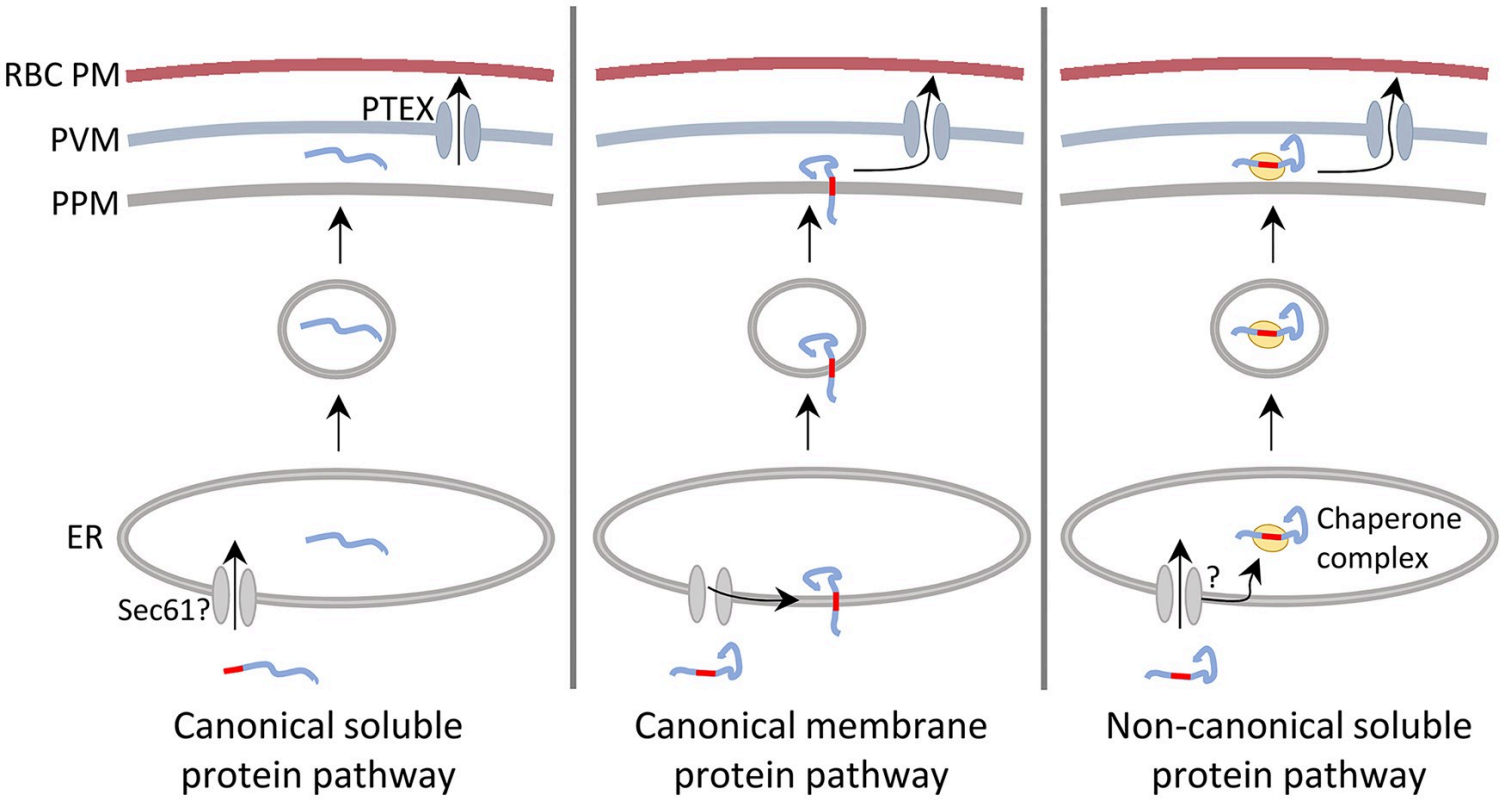
A model of the ways by which exported proteins can traverse the parasite secretory pathway. RBC PM, red blood cell plasma membrane; PVM, parasitophorous vacuole membrane; PPM, parasite plasma membrane; ER, endoplasmic reticulum. Hydrophobic targeting sequences (signal sequences or transmembrane segments) are shown in red.

Overall, our data together with that of others, suggest that there are three distinct means by which exported proteins can traverse the parasite secretory pathway before being exported into the red blood cell. Firstly, soluble proteins are targeted to the ER lumen by an N-terminal hydrophobic segment that may be cleaved by either plasmepsin V or signal peptidase [10,12,14]. As these proteins lack other hydrophobic segments that can mediate ER-targeting or membrane integration, they would be translocated entirely into the ER lumen by the Sec61 complex. Subsequently, the proteins would be trafficked to the parasitophorous vacuole by vesicular transport (likely in a complex with HSP101 [22]), unfolded and transported into the red blood cell by the PTEX complex (see canonical soluble pathway; Fig 7).

The second pathway would mediate export of proteins that contain internal non-cleaved hydrophobic segments that integrate into the ER membrane (canonical membrane protein pathway; Fig 7). Having partitioned into the ER membrane, these proteins would be trafficked to the parasite plasma membrane as integral membrane proteins, where they would be membrane-extracted and then translocated into the red blood cell by the PTEX complex. Membrane extraction and translocation may be mechanistically connected; it is unclear if an additional protein-conducting channel, residing in the parasite plasma membrane, would be required for the membrane extraction step. In support of this model, REX2 has a propensity to integrate into the ER membrane when translated *in vitro*, in the presence of rat liver ER-microsomes [23]. Additionally, fusion of REX2 to tightly folded protein domains, DHFR or BPTI, leads to arrest of the protein in either the parasite plasma membrane or the parasitophorous vacuole membrane, respectively, suggesting that these proteins are extracted from these membranes [23,24]. STEVOR proteins have also been shown to be efficiently exported when an SDEL sequence is added to their C-terminus, suggesting that they may also be integrated into the parasite ER membrane [38].

The third export-pathway mediates export of proteins such as Pf332, which are targeted to the ER by an internal, non-cleaved hydrophobic segment that either does not partition laterally into the ER lipid bilayer during translocation through the Sec61 channel, or is integrated and rapidly extracted from the ER membrane. These proteins would form non-integrated species within the ER lumen. As exposed hydrophobic residues would make the proteins aggregation prone, it is likely that the hydrophobic segment would have to be maintained in a soluble state by engaging with chaperone proteins within the ER lumen. The soluble chaperone-protein complexes could then be trafficked down the secretory pathway (non-canonical soluble pathway; Fig 7). Fractionation experiments suggest that PfEMP1 proteins also use such a pathway, forming non-integrated species both in the ER and parasitophorous vacuole [21,25].

In our current experiments, an SDEL sequence leads to partial retention of both SBP1 and MAHRP2 in the ER. The fraction of the SBP1 and MAHRP2 proteins that is retained in the ER by the SDEL sequence is likely trafficked using the non-canonical soluble pathway. Although integration of a fraction of these proteins into the membrane is the most plausible means by which these SDEL-tagged proteins could partially evade SDEL-mediated retention, it is also possible that they form a lumenal species that tightly associates with a chaperone that is actively trafficked down the secretory pathway and can thus be partially removed from the ER-recycling compartment [58]. Although, in this context it is difficult to explain the observation that export of soluble proteins is completely blocked by an SDEL sequence [36,38,48], interaction with the putative chaperone could be unique to membrane proteins. Alternatively, a transmembrane segment could enhance the affinity of the putative chaperone for an exported protein. This would be consistent with the observation that a transmembrane segment can enhance export of a model exported protein in parasites expressing an HSP101 protein whose function is partially compromised [59]. Notably, SBP1, when fused to a tightly folded DHFR domain, becomes arrested in the parasitophorous vacuole rather than the parasite plasma membrane [24]. This would be consistent with a model in which the protein inserts into the ER co-translationally (which would likely prevent DHFR folding within the cytoplasm). Once released within the ER, the DHFR domain might fold and this ultimately would prevent translocation of the protein through the PTEX complex in the parasitophorous vacuole membrane. Although we have attempted to perform similar experiments with Pf332-mDHFR, extensive proteolysis of the protein precludes a clear interpretation.

A number of unconventional secretion pathways have been characterised in eukaryotic cells, including direct translocation of proteins across the plasma membrane and secretion of autophagosomes or other organelles [60,61]. However, proteins that utilise these pathways typically lack a signal sequence or transmembrane segment. Exported proteins either contain an N-terminal signal sequence-like sequence, a transmembrane-like sequence, or both, suggesting that they do not utilise these unconventional secretion pathways i.e. they do not have the characteristics of proteins known to use these pathways. Although the exact trafficking pathway taken by exported proteins from the ER to the plasma membrane is poorly understood, many lines of evidence indicate that the ER is the starting point for protein export [12–15,36,62].

Typically, internal transmembrane-like sequences mediate membrane integration [63] so it remains unclear how proteins such as Pf332 reach the lumen of the ER. Either the protein does not partition laterally into the bilayer during translocation into the ER, or is integrated and efficiently extracted from the ER membrane. Although it is not known whether specific Sec61 complexes are utilised by many exported proteins including Pf332, at least a subset of PEXEL proteins use the post-translational ER-translocation pathway which involves a complex of Sec61-62-63. In parasites, this complex can also associate with a *Plasmodium*-specific complex comprising SPC25 and plasmepsin V [30]. In structures of the yeast Sec61-62-63 complex it is evident that two Sec62 transmembrane segments and the intervening ER-lumenal loop pass in front of the Sec61 lateral exit. This might, in some channel conformations, occlude the pathway into the bilayer and thus increase the propensity of hydrophobic polypeptides to translocate across, rather than partition into the membrane [64,65]. Although this point is speculative, it suggests that proteins such as Sec62 or *Plasmodium*-specific proteins (such as plasmepsin V or others) might occlude the Sec61-lateral exit. Although a role for Sec61 in ER-translocation of a subset of PEXEL-containing proteins is experimentally supported [30], and it is likely that a post- or co-translational Sec61-mediated pathway is used by other exported proteins such as PNEPs, it is difficult to completely rule out use of other translocases.

Exported proteins that have internal hydrophobic segments would likely aggregate within the ER lumen unless associated with protein chaperones. The nature of the putative chaperone(s) remains unclear. However, recent experiments show that a significant fraction of the HSP101 protein resides in the parasite ER [22,66,67]. It is proposed that HSP101 interacts with nascent-exported proteins as they emerge into the ER and may then escort them to the parasitophorous vacuole, where the complex could engage with PTEX150 and the channel protein EXP2. In this model, unfolding and translocation through the EXP2 channel would be followed by dissociation and recycling of the HSP101 back to the ER [22].

In conclusion, many aspects of protein trafficking in the malaria parasite are unique and essential for parasite survival and virulence [27,28]. This suggests that, in addition to export-related proteins such as plasmepsin V and HSP101, there may be other *Plasmodium*-specific factors that facilitate trafficking and represent suitable targets for generation of novel anti-malarial drugs.

## Methods

### Ethics statement

The use of human blood for experiments described here was approved by the National Health Service National Research Ethics Service, UK, East London REC3 committee (Research Ethics Committee reference 10H/H0701/121).

### Parasite culture and transfection

Parasites were cultured in human red blood cells (NHS-BT) in 5% oxygen, 5% carbon dioxide, 90% nitrogen. Transfection of protein expression plasmids into 3D7 AttB parasites [68] or derivatives, was performed using a modification of a previously published method [69]. Cytomix-washed red blood cells, 100 μg of a plasmid (derived from pINT [68]) for expression of the Bxb1 integrase [70], and 100 μg of protein expression plasmid (derivative of pA221 that contains an AttP site and blasticidin selection marker [46]), were mixed and electroporated using a BioRAD Gene Pulser electroporator. Asynchronous parasites were added to the electroporated red blood cells. After one day, parasites were selected with WR99210 (2.5 nM) and blasticidin (2 μg/ml). All proteins, except GFP_1-10_ constructs (see below), were expressed using plasmids allowing integration into an AttB site.

For expression of GFP_1-10_ fragments either in the parasite cytoplasm or ER lumen, expression cassettes were integrated into the *pfs47* gene in 3D7 AttB parasites. A cassette comprising the HSP86 promoter and 3’ UTR of *pbDT* was used to express GFP_1-10_ fragments. This cassette was flanked by homology regions derived from the *pfs47* gene and flanking sequences. These plasmids were constructed in pBluescript and also contained a cassette for expression of the negative selection marker YFCU. However, the YFCU open reading frame was disrupted when plasmids were linearised prior to transfection. Cytoplasmic GFP_1-10_ corresponds to GFP_1-10_ with a C-terminal Strep-tag and was assembled in plasmid pA827. ER-lumenal GFP_1-10_ corresponds to the signal peptide from PF3D7_0208500 fused to GFP_1-10_ sequence followed by a Strep-tag and the final 11 residues of PfBiP which includes the SDEL sequence (assembled in plasmid pA826). A guide RNA targeting the *pfs47* gene (AATGTTAAGCCAACTGTAGT) [71] and Cas9 were expressed from plasmid pA856. This plasmid is a modification of plasmid pDC2-cam-Cas9-U6-chRNA-hDHFR [72]; the DHFR encoding gene was replaced by a puromycin acetyl-transferase coding sequence that was followed by a sequence encoding a T2A peptide and the negative selection marker YFCU. Ring stage parasites were transfected with plasmids pA856 and either pA826 or pA827. pA826-7 were first linearized using BamH1 and Xba1. After transfection of ring-stage parasites using a BioRAD Gene Pulser electroporator, parasites were selected for 10 days with puromycin and WR99210. Parasites were then grown without puromycin for at least 20 days followed by negative selection using 5-fluorocytosine (1 μM) for at least 10 days. Parasites were cloned by dilution [73], and insertion of the expression cassette into the *pfs47* gene was confirmed by PCR (S9 Fig). Phusion polymerase was used for PCR using primers 1 and 2 (taattgcatacacataaatatttgtgttgtac and ggagataaatgtaaggtaaatatacacaaac). Thirty PCR cycles were performed comprising the following steps; 98°C for 15 seconds, 50°C for 30 seconds and 60°C for 4 minutes.

### Western blotting

Parasites were Percoll purified and approximately 10^6^ parasites were loaded per lane. Gels were transferred to nitrocellulose and probed with anti-GFP (Torrey Pines, TP401), anti-RFP (MBL, PM005), or anti-HA (Invitrogen, 326700), followed by anti-rabbit DyLight680 antibody (Thermo Scientific, 35568), or anti-mouse DyLight800 (Thermo Scientific, SA5-10172). Blots were imaged using a LI-COR Odyssey CLx imaging system.

### Imaging

Parasites were passaged or medium was replaced with fresh complete medium on the two days preceding imaging. Parasites were prepared by placing approximately 3–5 μl of culture material between a slide and coverslip. Images were acquired using Zen 2012 software and a Zeiss Observer Z1. Figures were prepared using Image J [74]. Images shown in all figures were acquired using an 800 ms exposure for GFP and mCherry, or a 10 ms exposure for Hoechst. For all mCherry and GFP figure panels in the main text the brightness ranges used were 1–800 and 1–1000, respectively.

For imaging Brefeldin A-treated parasites, asynchronous cultures were used but only young trophozoite-stage parasites were imaged. Parasites were treated with 1 μg/ml of Brefeldin A for 4 hours (Brefeldin A was added from a stock solution dissolved in DMSO). Imaging was performed between 4 and 5 hours after addition of Brefeldin A.

### Quantitation of fluorescence images

Trophozoite-stage parasites, containing a visible food vacuole but estimated to occupy less than 50% of the red blood cell, were randomly selected for imaging and focused in a phase contrast view. For each channel, a Z-stack comprising seven images was acquired with 200 nm between slices. Images of parasites were acquired using 800 ms exposures for both red and green channels. For each channel, the stack of seven images was combined into separate sum projections, using ImageJ [74]. In the phase contrast image, polygons outlining the parasite and encompassing erythrocyte were manually defined. Additionally, two squares were placed within two different uninfected erythrocytes for the purpose of measuring background fluorescence. All shapes were transferred to the fluorescence sum projection images to measure fluorescence intensity and background. Total fluorescence within the polygon delineating the red blood cell plasma membrane (this includes fluorescence within the parasite) was measured, and after subtraction of background fluorescence, is referred to as ‘total fluorescence’. Fluorescence within the polygon outlining the parasite was determined similarly. These values were used to determine the fraction of red fluorescence retained within the parasite (fluorescence in the parasite/total fluorescence). For comparison of mCherry and GFP fluorescence levels ‘total fluorescence’ values were used for both channels. Data were plotted and P-values determined using GraphPad Prism.

### Immunofluorescence labelling

Parasites were allowed to settle on poly-lysine coated coverslides for 20 minutes and fixed in 4% paraformaldehyde in PBS for 30 minutes. Coverslides were incubated for 15 minutes in 100 mM glycine in PBS, 10 minutes in 0.1% Triton X100, followed by 3% bovine serum albumin (BSA) in PBS for 30 minutes. Parasites were labelled for 30 minutes with rabbit anti-MAHRP1 antibody [75], mouse anti-plasmepsin V antibody [76], or rabbit anti-GFP antibodies [77], diluted in PBS containing 3% BSA. After washing, slides were incubated with labelled secondary antibodies (Invitrogen) for 30 minutes.

### Mass spectrometry

Parasites expressing REX3_RQLSE_:Pf332:_C-S11_:SDEL were lysed in 50 mM Tris pH 8, 200 mM NaCl, 0.1 mM EDTA, 1% TX100, 0.1% SDS. Lysates were clarified by centrifugation at 4000 rpm for 10 minutes (A481 Eppendorf rotor) and then incubated for 1 hour with Streptactin beads. After washing, proteins were eluted with 10 mM Biotin in lysis buffer. Eluted proteins from two experiments were run on a 12% gel, stained with Coomassie blue, and the band corresponding to REX3_RQLSE_:Pf332:_C-S11_:SDEL was excised for tryptic digestion (S2 Text). For a third experiment, the proteins were eluted from the Streptactin beads and trypsin-digested directly.

### Sodium carbonate extraction

Parasites were percoll purified from an asynchronous culture and washed in PBS. 10^7^ parasites were pelleted and after removal of the supernatant resuspended in 200 μl of 5 mM Tris pH 8 containing 20 μg/ml DNAse I and protease inhibitor cocktail (Roche). After incubation on ice for 10 minutes the samples were centrifuged at 65,000 rpm in a Beckman TLA120.1 rotor. The pellet was resuspended in 200 μl 0.1M sodium carbonate pH 11.5. After incubation on ice for 30 minutes the resuspended pellets were centrifuged at 65,000 rpm. The supernatant was removed and the pellet resuspended before addition of SDS-sample buffer. Before addition of SDS sample buffer to the initial supernatant obtained after hypotonic lysis in 5 mM Tris, centrifugation was repeated to remove any residual membranes. Equivalent volumes of samples were analysed by SDS-PAGE and western blotting using anti-RFP (MBL, PM005), anti-plasmepsin V [76], and rabbit anti-SERA5 [78] antibodies.

## Supporting information

S1 Fig Western blotting analysis of Pf332-expressing parasites. (A) Western blots of parasites expressing the indicated proteins are shown. Blots were probed with anti-mCherry. (B) Western blots of parasites for comparison of expression levels of the indicated Pf332 proteins. The blots were probed with anti-mCherry (shown in red) and anti-plasmepsin V as a loading control (shown in green). (C) Western blots of parasites expressing the indicated proteins are shown. Blots were probed with anti-mCherry or anti-GFP as indicated.
(TIF)

S2 Fig Immunofluorescence and live-cell microscopy of Pf332-expressing parasites. (A-D) Immunofluorescence labelling of parasites expressing the indicated mCherry tagged proteins. Intrinsic mCherry fluorescence of the proteins is shown in red. Labelling with anti-MAHRP1 or anti-plasmepsin V is shown in green. (E) Immunofluorescence labelling of parasites expressing ER-lumenal GFP_1-10_ only. Parasites were labelled with anti-GFP (red) and anti-plasmepsin V (green). (F) Phase contrast and fluorescence images of parasites expressing the Pf332:Int-mCherry:_C-S11_:DSLE proteins either alone or with the indicated GFP_1-10_ proteins. Scale bar: 2 μm.
(TIF)

S3 Fig Fluorescence microscopy analysis of split-GFP-expressing parasites. (A-B) Cartoon representation of plasmepsin V with a C-terminal S11 tag (plasmepsinV:3xHA:_C-S11_), and phase contrast and green fluorescence images of parasites expressing GFP_1-10_ fragments together with plasmepsinV:3xHA:_C-S11_ are shown. (C-D) Images of parasites co-expressing cytoplasmic mCherry that has a C-terminal S11 tag with either ER-lumenal GFP_1-10_ or cytoplasmic GFP_1-10_ are shown. For increased clarity and comparison to figures in the main text, two brightness ranges are shown for each image, as indicated. For GFP and mCherry images in the main text brightness settings of 0–1000 and 0–800 were used, respectively. In the images shown here, 0–1000 and 0–800 are shown for GFP and mCherry, respectively, but a brightness setting of 0–4095 is also shown for both channels. (E-F) Images of parasites co-expressing ER-lumenal mCherry (ER-lumenal mCherry comprises the N-terminal signal peptide derived from PF3D7_0827900, mCherry, a C-terminal S11 tag, and a STREP tag, followed by an SDEL sequence) with either ER-lumenal GFP_1-10_ or cytoplasmic GFP_1-10_, are shown.
(TIF)

S4 Fig Western blot analysis of REX3:Pf332 expressing parasites and sodium carbonate extraction experiments of Pf332, SBP1 and MAHRP2-expressing parasites. (A)Western blots of parasites expressing the indicated REX3:Pf332 fusion proteins are shown. Blots were probed with anti-mCherry or anti-GFP antibodies as indicated. (B) Sodium carbonate extraction of Pf332:mCherry:DSLE and Pf332:mCherry:SDEL. Parasites were hypotonically lysed. The membrane fraction was then extracted with sodium carbonate. Equivalent volumes of each fraction are loaded. Blots were probed with anti-mCherry for detection of the Pf332 proteins. Plasmepsin V and SERA5 were used as markers of the membrane and soluble fractions, respectively. (C) Sodium carbonate extraction of mCherry tagged SBP1:_C-S11_:DSLE and MAHRP2:_C-S11_:DSLE. Blots were prepared and probed as in (B).
(TIF)

S5 Fig Analysis of SBP1 parasites. (A) Western blots of parasites expressing the indicated SBP1 proteins are shown. Blots were probed with anti-mCherry or anti-GFP antibodies as indicated. (B) Western blot of parasites for comparison of expression levels of the indicated SBP1 proteins. The blots were probed with anti-mCherry (shown in red) and anti-plasmepsin V as a loading control (shown in green). (C) Immunofluorescence labelling of parasites expressing mCherry tagged SBP1:_C-S11_:DSLE. Intrinsic mCherry fluorescence of the proteins is shown in red. Labelling with anti-MAHRP1 is shown in green. (D) Immunofluorescence labelling of parasites expressing mCherry tagged SBP1:_C-S11_:DSLE and treated with Brefeldin A. Intrinsic mCherry fluorescence of the proteins is shown in red. Labelling with anti-plasmepsin V is shown in green. (E) Phase contrast and fluorescence images of parasites expressing mCherry tagged SBP1:_C-S11_:DSLE and treated with DMSO are shown. Scale bar: 2 μm.
(TIF)

S6 Fig High contrast GFP images of SBP1-expressing parasites. (A-F) Phase contrast and fluorescence images of parasites expressing the indicated proteins are shown. Proteins were expressed alone, co-expressed with ER-lumenal GFP_1-10_ or cytoplasmic GFP_1-10_, as indicated. Images are identical to those in the main text Figs 4 and 5 except that high contrast images of the GFP channel are shown. Contrast settings for GFP images are set at 0–200 to show weak GFP signal. Scale bar: 2 μm.
(TIF)

S7 Fig Analysis of MAHRP2 parasites. (A)Western blots of parasites expressing the indicated MAHRP2 proteins are shown. Blots were probed with anti-mCherry or anti-GFP antibodies as indicated. (B)Western blot of parasites for comparison of expression levels of the indicated MAHRP2 proteins. The blots were probed with anti-mCherry (shown in red) and anti-plasmepsin V as a loading control (shown in green). (C) Immunofluorescence labelling of parasites expressing mCherry tagged MAHRP2:_C-S11_:DSLE. Intrinsic mCherry fluorescence of the proteins is shown in red. Labelling with anti-MAHRP1 is shown in green. (D) Immunofluorescence labelling of parasites expressing mCherry tagged MAHRP2:_C-S11_:DSLE and treated with Brefeldin A. Intrinsic mCherry fluorescence of the proteins is shown in red. Labelling with anti-plasmepsin V is shown in green. (E) Phase contrast and fluorescence images of parasites expressing mCherry tagged MAHRP2:_C-S11_:DSLE and treated with DMSO are shown. Scale bar: 2 μm.
(TIF)

S8 Fig High contrast GFP images of MAHRP2-expressing parasites. (A-C) Phase contrast and fluorescence images of parasites expressing the indicated proteins are shown. Proteins were expressed alone, co-expressed with ER-lumenal GFP_1-10_ or cytoplasmic GFP_1-10_, as indicated. Images are identical to those in the main text Fig 6 except that high contrast images of the GFP channel are shown. Contrast settings for GFP images are set at 0–200 to show weak GFP signal. Scale bar: 2 μm.
(TIF)

S9 Fig PCR analysis of split-GFP cassette integration into the pfs47 locus. (A) Diagram of the unmodified *pfs47* gene locus, a plasmid containing a GFP_1-10_ expression cassette, and the *pfs47* locus modified with a GFP_1-10_ expression cassette. The position targeted by the gRNA within the *pfs47* gene, regions of homology between the *pfs47* locus and the repair plasmid, and binding sites for primer 1 and primer 2 used for PCR analysis are shown. (B) PCR analysis of parasites with GFP_1-10_ expression cassettes integrated into the *pfs47* gene locus. PCR reactions were performed using genomic DNA from cloned parasites and primers 1 and 2 (taattgcatacacataaatatttgtgttgtac and ggagataaatgtaaggtaaatatacacaaac) and analysed using an ethidium bromide stained agarose gel.
(TIF)

S1 Table Transmembrane domain analysis. (DOCX)

S1 Text Characterisation of GFP_1-10_ protein expression in parasites. (DOCX)

S2 Text Mass spectrometry methods. (DOCX)

S1 Data Supplementary file containing raw data for Figs 5 and 6. (XLSX)
