# Supplementary figures and images for: Formation of ER-lumenal intermediates during export of Plasmodium proteins containing transmembrane-like hydrophobic sequences
Source: PLoS Pathog. 2023 Mar 31;19(3):e1011281. doi: 10.1371/journal.ppat.1011281 (PMC10096305; doi:10.1371/journal.ppat.1011281)

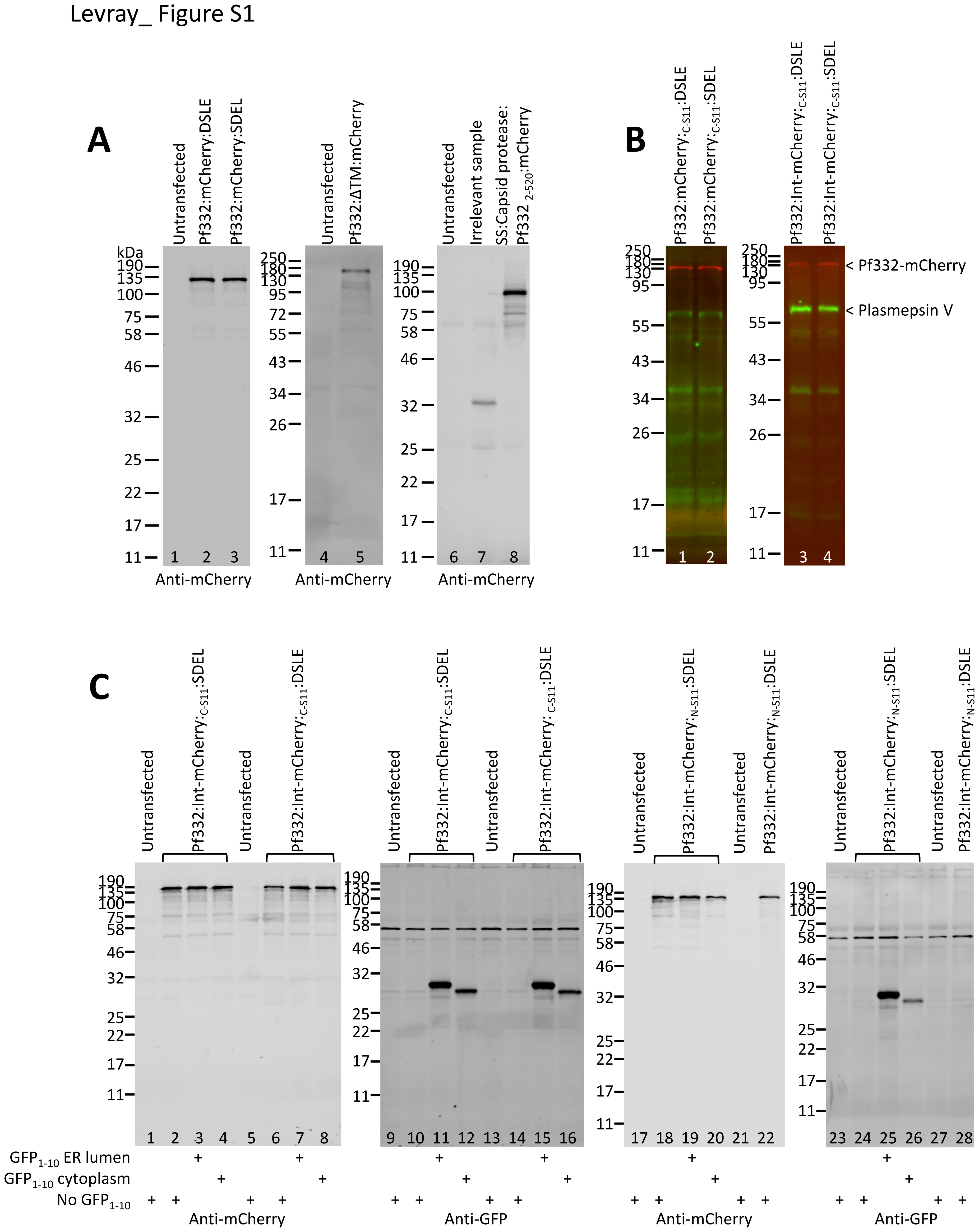

Supplement: S1 Fig — (A) Western blots of parasites expressing the indicated proteins are shown. Blots were probed with anti-mCherry. (B) Western blots of parasites for comparison of expression levels of the indicated Pf332 proteins. The blots were probed with anti-mCherry (shown in red) and anti-plasmepsin V as a loading control (shown in green). (C) Western blots of parasites expressing the indicated proteins are shown. Blots were probed with anti-mCherry or anti-GFP as indicated. (TIF) [file ppat.1011281.s001.tif]

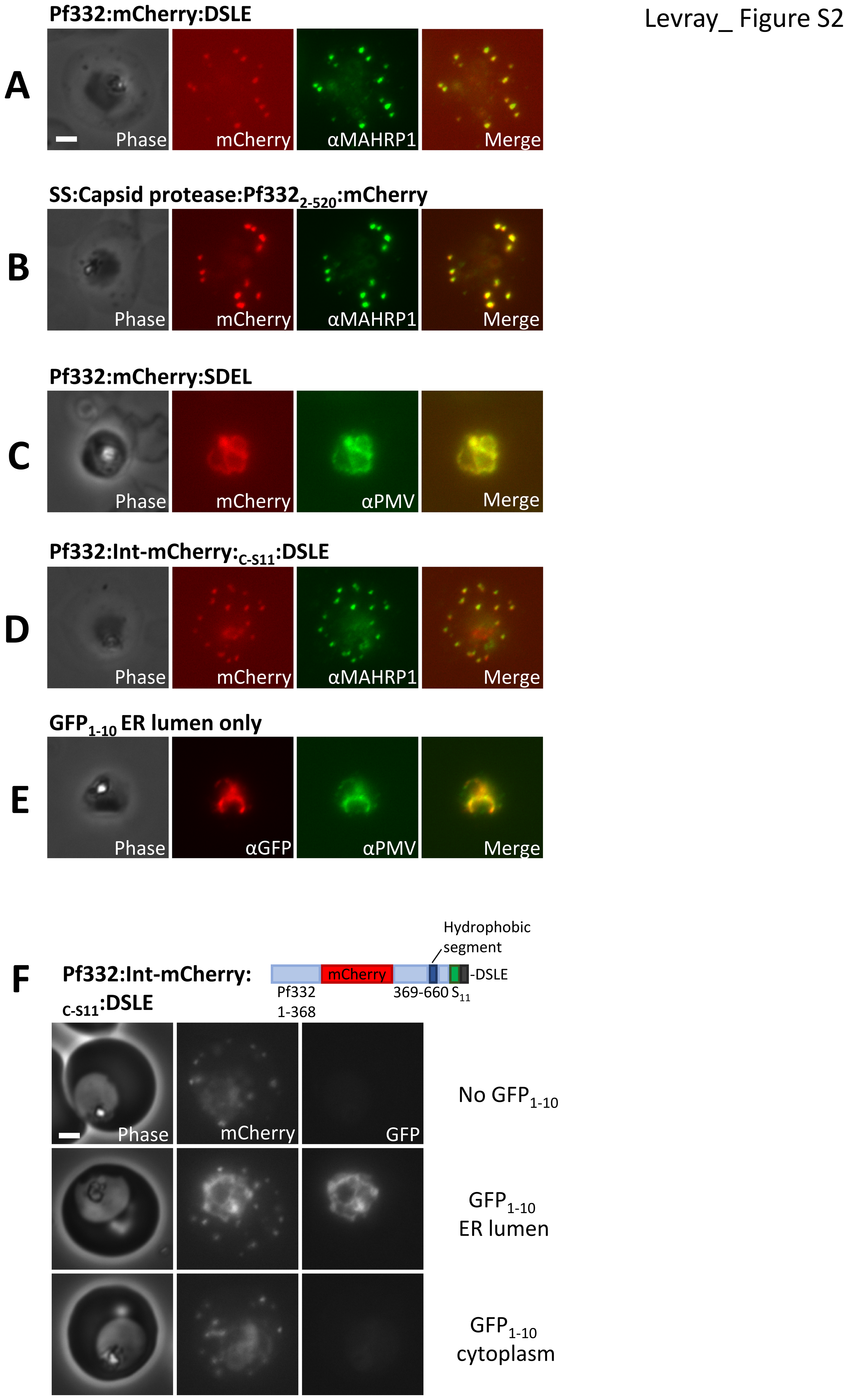

Supplement: S2 Fig — (A-D) Immunofluorescence labelling of parasites expressing the indicated mCherry tagged proteins. Intrinsic mCherry fluorescence of the proteins is shown in red. Labelling with anti-MAHRP1 or anti-plasmepsin V is shown in green. (E) Immunofluorescence labelling of parasites expressing ER-lumenal GFP1-10 only. Parasites were labelled with anti-GFP (red) and anti-plasmepsin V (green). (F) Phase contrast and fluorescence images of parasites expressing the Pf332:Int-mCherry:C-S11:DSLE proteins either alone or with the indicated GFP1-10 proteins. Scale bar: 2 μm. (TIF) [file ppat.1011281.s002.tif]

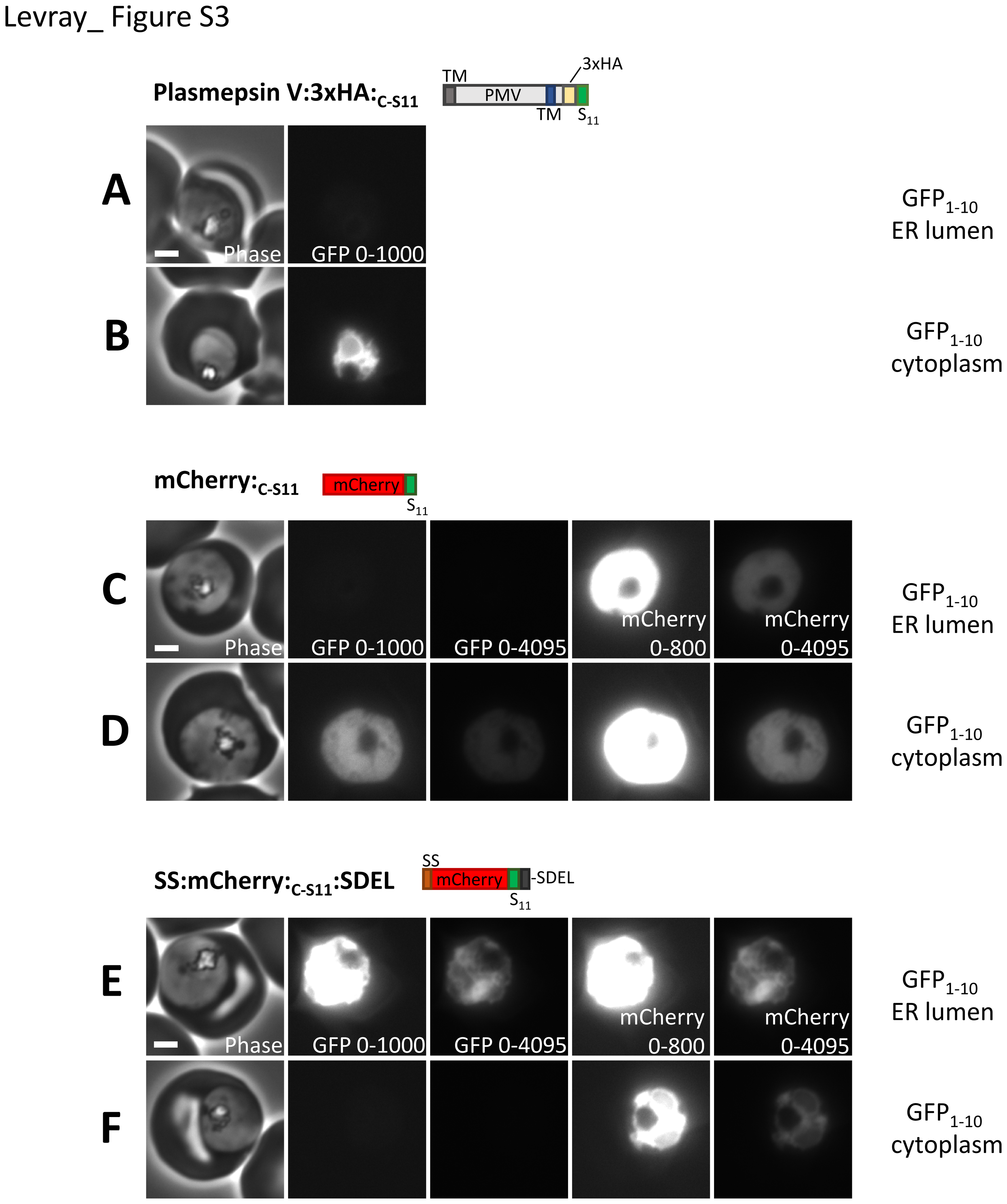

Supplement: S3 Fig — (A-B) Cartoon representation of plasmepsin V with a C-terminal S11 tag (plasmepsinV:3xHA:C-S11), and phase contrast and green fluorescence images of parasites expressing GFP1-10 fragments together with plasmepsinV:3xHA:C-S11 are shown. (C-D) Images of parasites co-expressing cytoplasmic mCherry that has a C-terminal S11 tag with either ER-lumenal GFP1-10 or cytoplasmic GFP1-10 are shown. For increased clarity and comparison to figures in the main text, two brightness ranges are shown for each image, as indicated. For GFP and mCherry images in the main text brightness settings of 0–1000 and 0–800 were used, respectively. In the images shown here, 0–1000 and 0–800 are shown for GFP and mCherry, respectively, but a brightness setting of 0–4095 is also shown for both channels. (E-F) Images of parasites co-expressing ER-lumenal mCherry (ER-lumenal mCherry comprises the N-terminal signal peptide derived from PF3D7_0827900, mCherry, a C-terminal S11 tag, and a STREP tag, followed by an SDEL sequence) with either ER-lumenal GFP1-10 or cytoplasmic GFP1-10, are shown. (TIF) [file ppat.1011281.s003.tif]

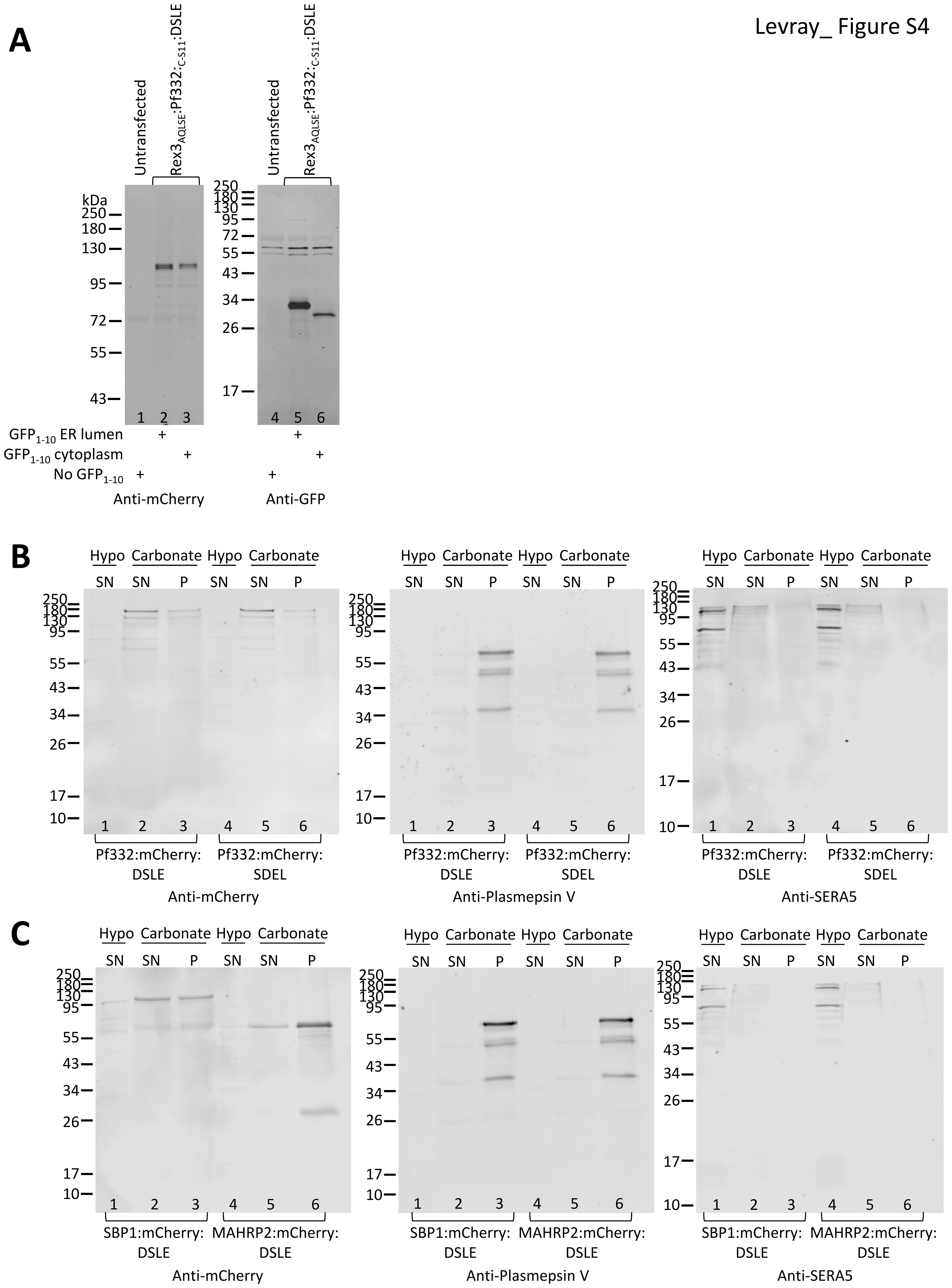

Supplement: S4 Fig — (A)Western blots of parasites expressing the indicated REX3:Pf332 fusion proteins are shown. Blots were probed with anti-mCherry or anti-GFP antibodies as indicated. (B) Sodium carbonate extraction of Pf332:mCherry:DSLE and Pf332:mCherry:SDEL. Parasites were hypotonically lysed. The membrane fraction was then extracted with sodium carbonate. Equivalent volumes of each fraction are loaded. Blots were probed with anti-mCherry for detection of the Pf332 proteins. Plasmepsin V and SERA5 were used as markers of the membrane and soluble fractions, respectively. (C) Sodium carbonate extraction of mCherry tagged SBP1:C-S11:DSLE and MAHRP2:C-S11:DSLE. Blots were prepared and probed as in (B). (TIF) [file ppat.1011281.s004.tif]

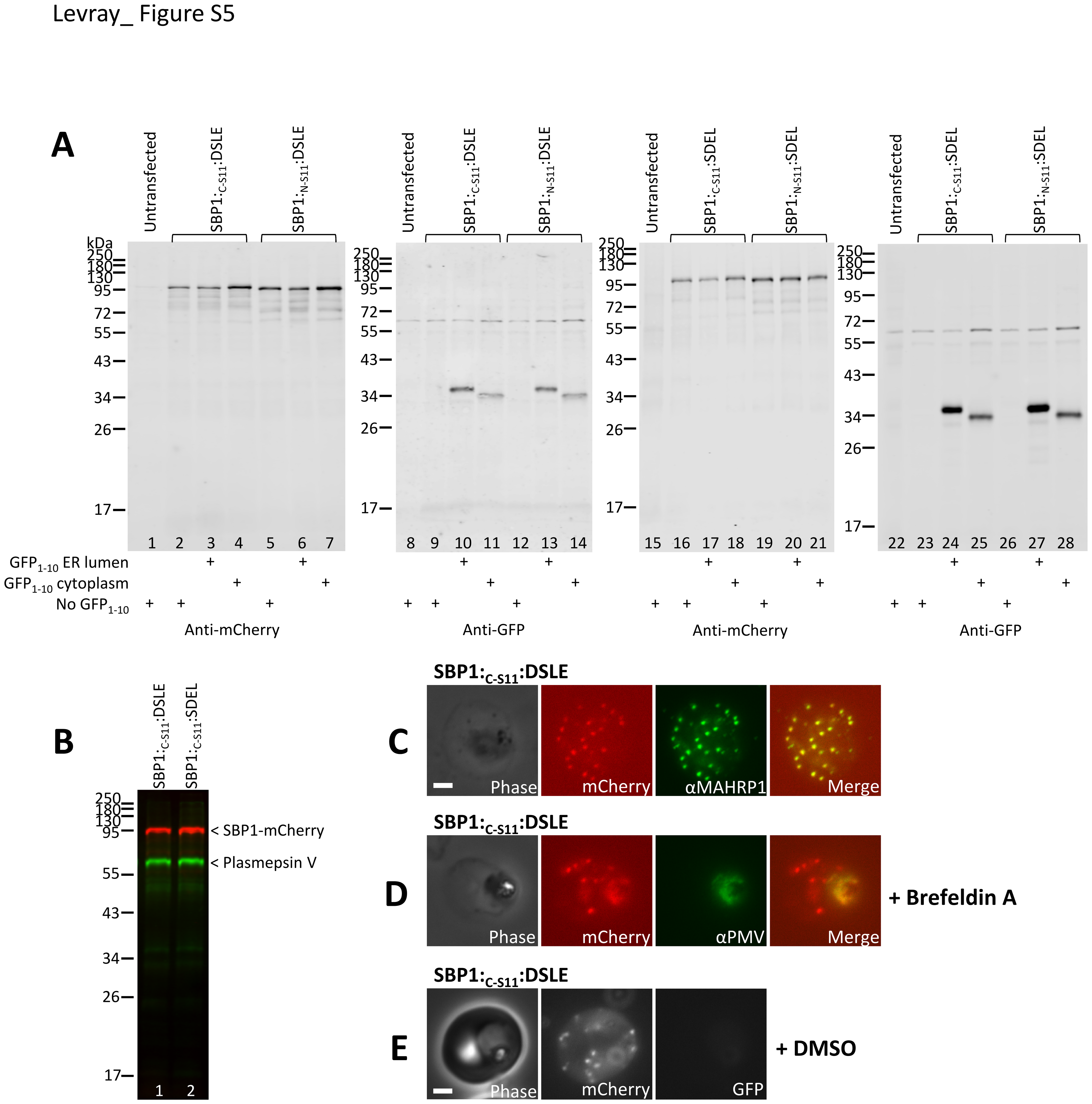

Supplement: S5 Fig — (A) Western blots of parasites expressing the indicated SBP1 proteins are shown. Blots were probed with anti-mCherry or anti-GFP antibodies as indicated. (B) Western blot of parasites for comparison of expression levels of the indicated SBP1 proteins. The blots were probed with anti-mCherry (shown in red) and anti-plasmepsin V as a loading control (shown in green). (C) Immunofluorescence labelling of parasites expressing mCherry tagged SBP1:C-S11:DSLE. Intrinsic mCherry fluorescence of the proteins is shown in red. Labelling with anti-MAHRP1 is shown in green. (D) Immunofluorescence labelling of parasites expressing mCherry tagged SBP1:C-S11:DSLE and treated with Brefeldin A. Intrinsic mCherry fluorescence of the proteins is shown in red. Labelling with anti-plasmepsin V is shown in green. (E) Phase contrast and fluorescence images of parasites expressing mCherry tagged SBP1:C-S11:DSLE and treated with DMSO are shown. Scale bar: 2 μm. (TIF) [file ppat.1011281.s005.tif]

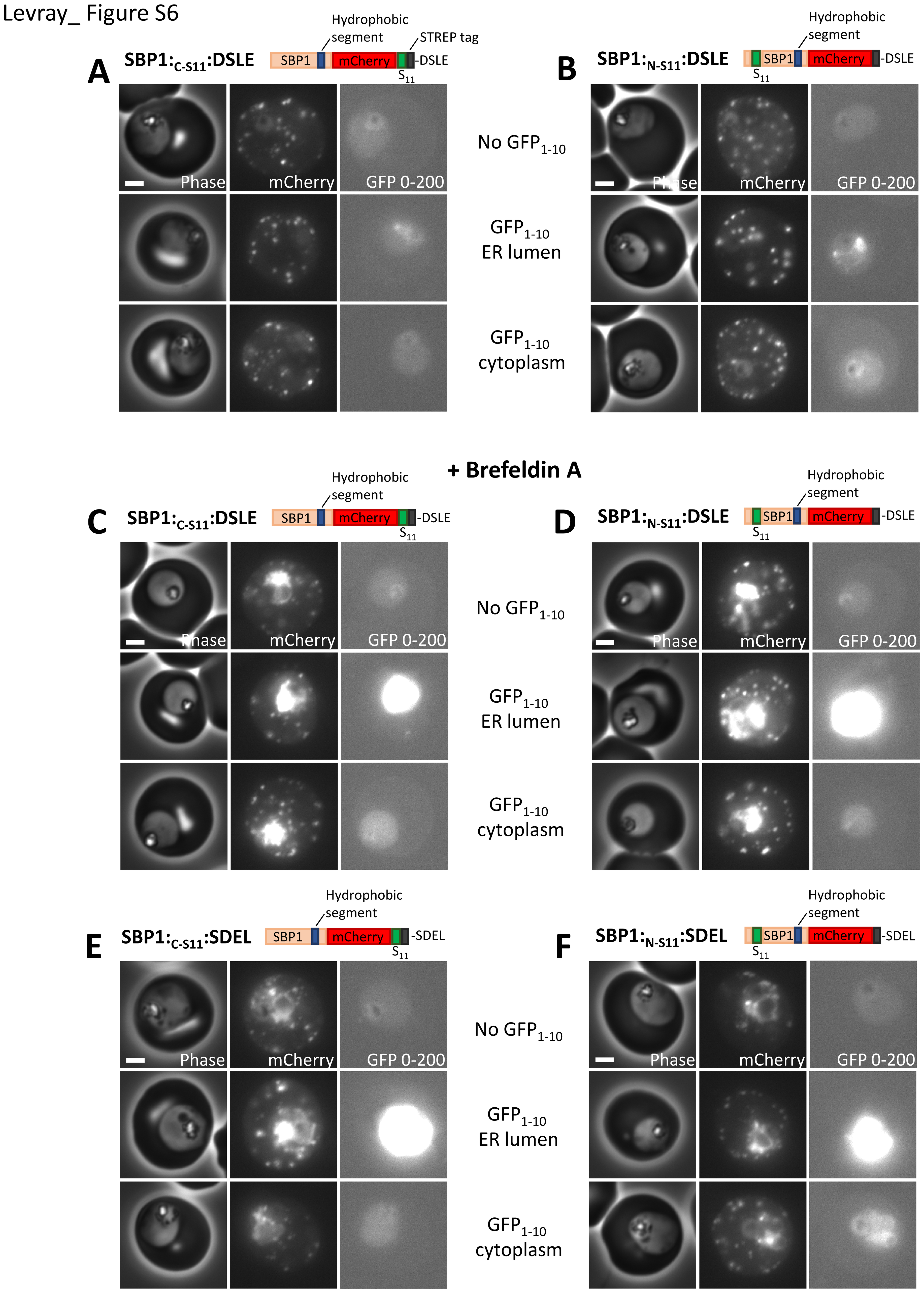

Supplement: S6 Fig — (A-F) Phase contrast and fluorescence images of parasites expressing the indicated proteins are shown. Proteins were expressed alone, co-expressed with ER-lumenal GFP1-10 or cytoplasmic GFP1-10, as indicated. Images are identical to those in the main text Figs 4 and 5 except that high contrast images of the GFP channel are shown. Contrast settings for GFP images are set at 0–200 to show weak GFP signal. Scale bar: 2 μm. (TIF) [file ppat.1011281.s006.tif]

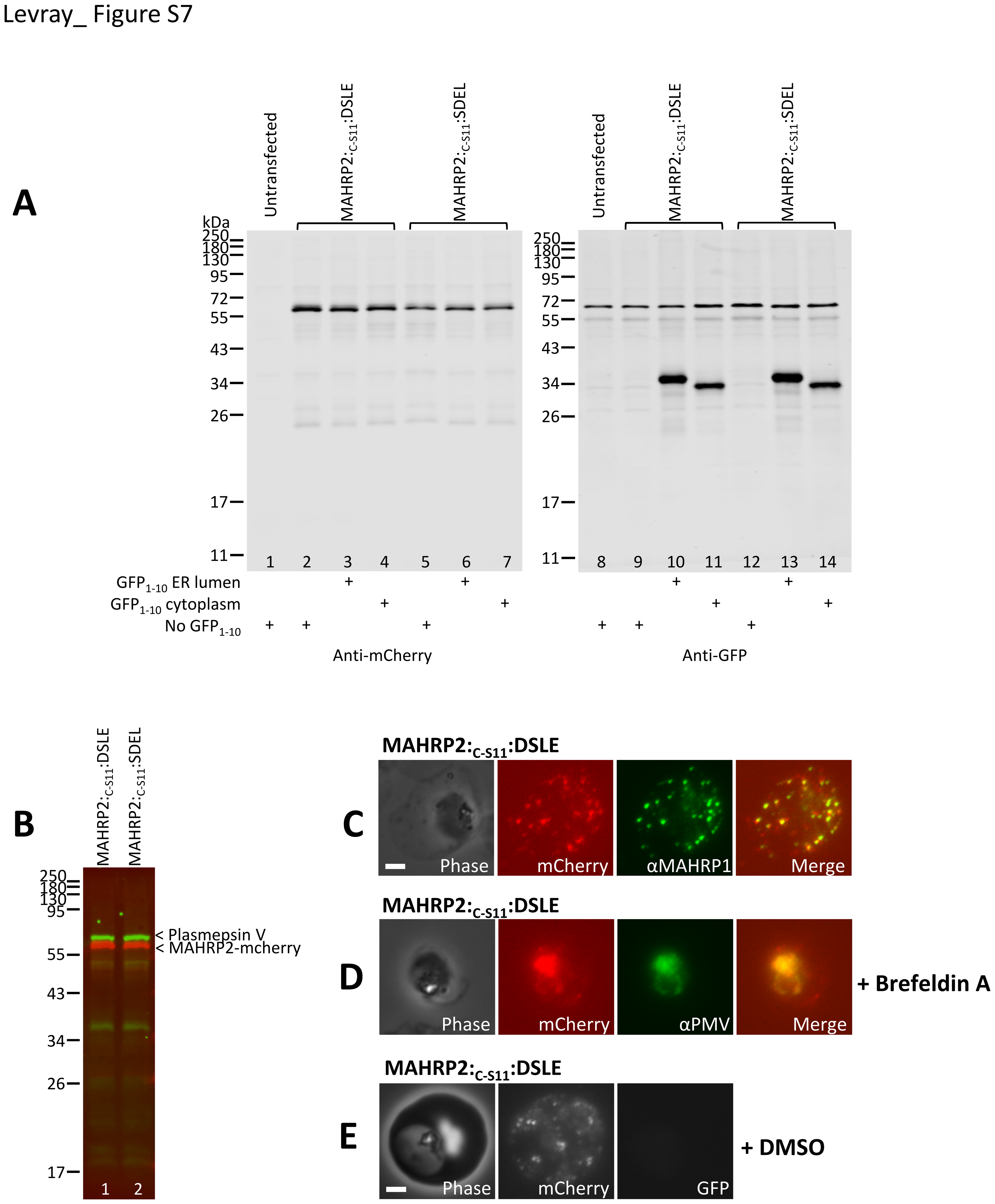

Supplement: S7 Fig — (A)Western blots of parasites expressing the indicated MAHRP2 proteins are shown. Blots were probed with anti-mCherry or anti-GFP antibodies as indicated. (B)Western blot of parasites for comparison of expression levels of the indicated MAHRP2 proteins. The blots were probed with anti-mCherry (shown in red) and anti-plasmepsin V as a loading control (shown in green). (C) Immunofluorescence labelling of parasites expressing mCherry tagged MAHRP2:C-S11:DSLE. Intrinsic mCherry fluorescence of the proteins is shown in red. Labelling with anti-MAHRP1 is shown in green. (D) Immunofluorescence labelling of parasites expressing mCherry tagged MAHRP2:C-S11:DSLE and treated with Brefeldin A. Intrinsic mCherry fluorescence of the proteins is shown in red. Labelling with anti-plasmepsin V is shown in green. (E) Phase contrast and fluorescence images of parasites expressing mCherry tagged MAHRP2:C-S11:DSLE and treated with DMSO are shown. Scale bar: 2 μm. (TIF) [file ppat.1011281.s007.tif]

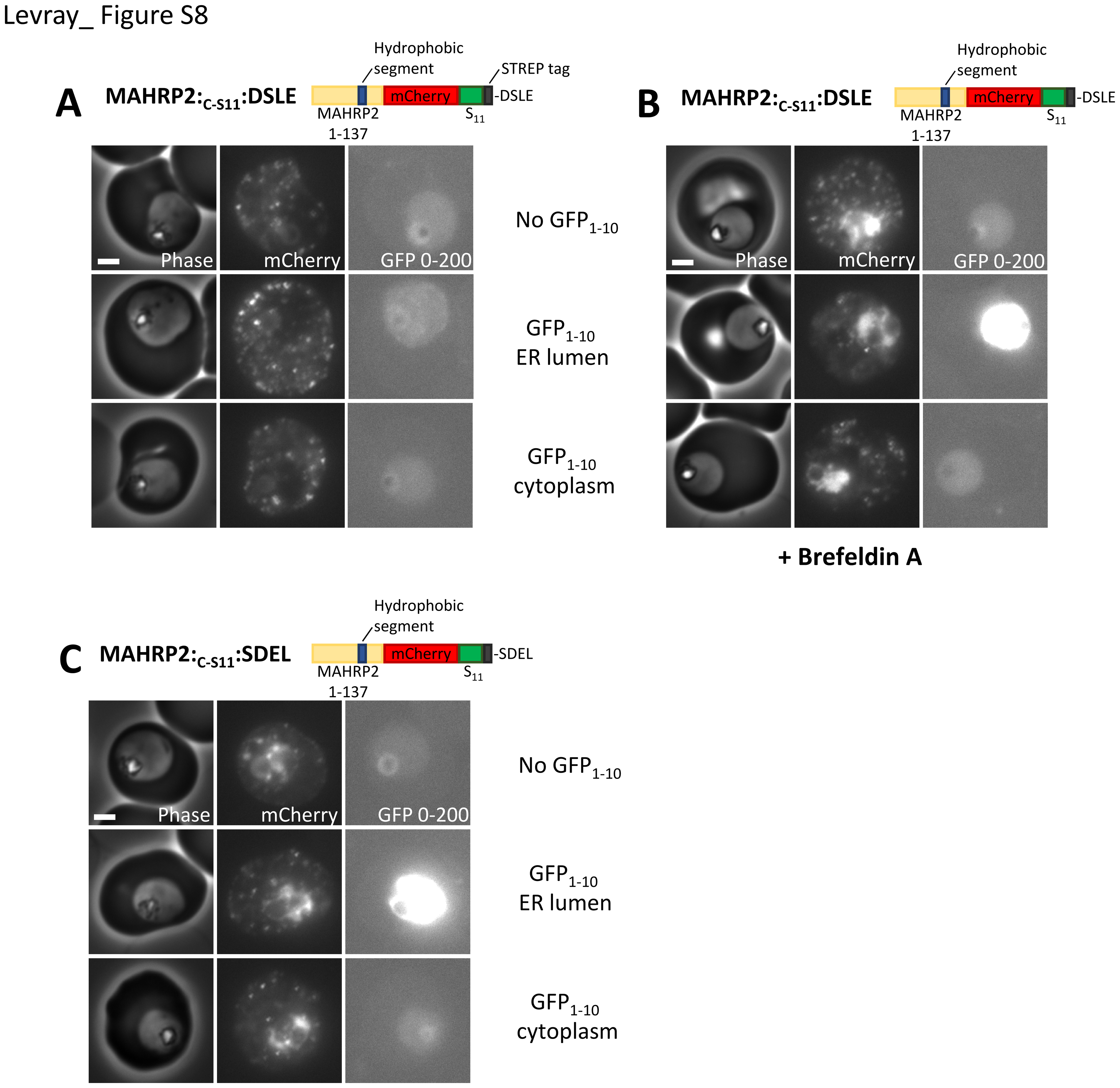

Supplement: S8 Fig — (A-C) Phase contrast and fluorescence images of parasites expressing the indicated proteins are shown. Proteins were expressed alone, co-expressed with ER-lumenal GFP1-10 or cytoplasmic GFP1-10, as indicated. Images are identical to those in the main text Fig 6 except that high contrast images of the GFP channel are shown. Contrast settings for GFP images are set at 0–200 to show weak GFP signal. Scale bar: 2 μm. (TIF) [file ppat.1011281.s008.tif]

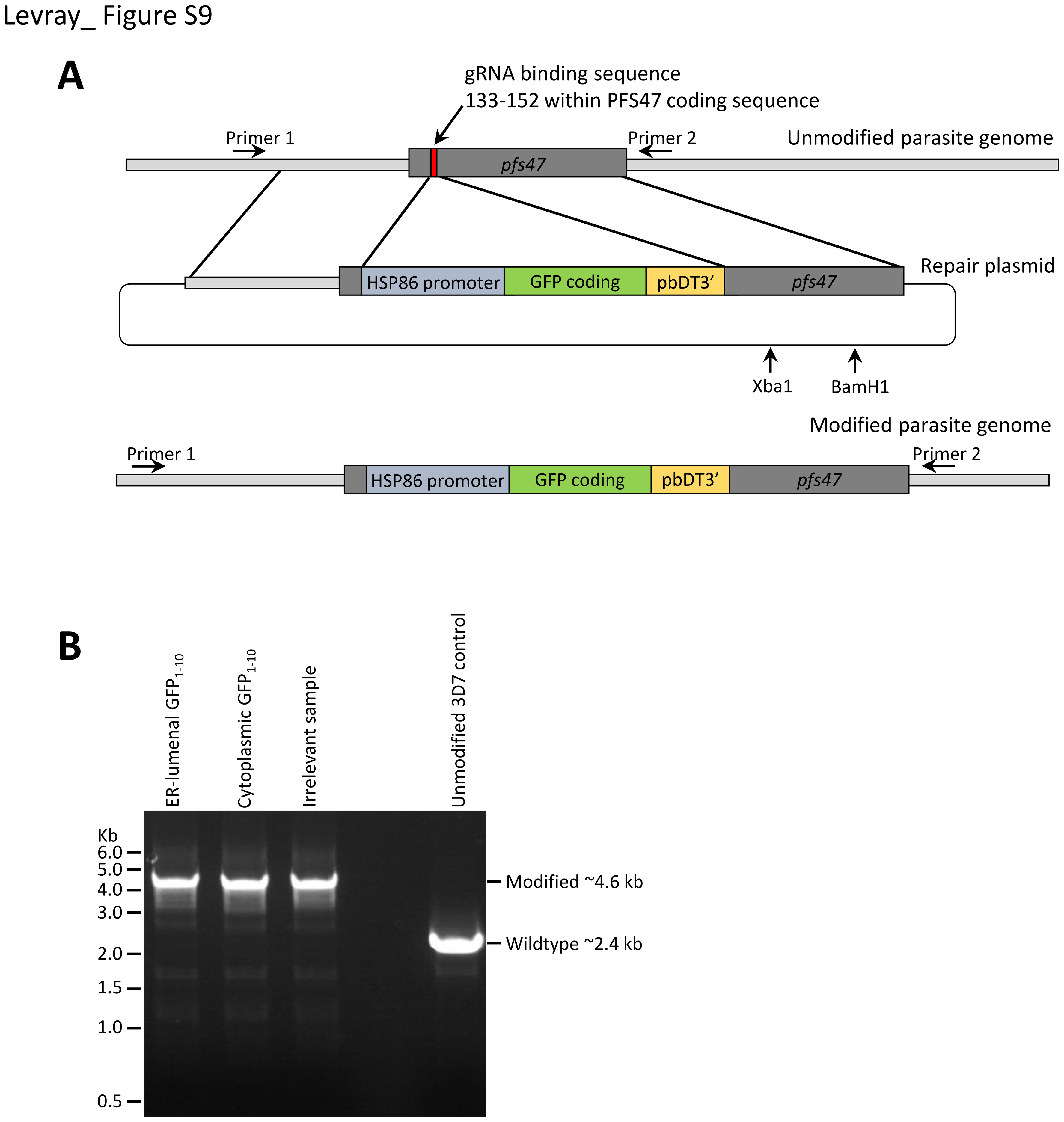

Supplement: S9 Fig — (A) Diagram of the unmodified pfs47 gene locus, a plasmid containing a GFP1-10 expression cassette, and the pfs47 locus modified with a GFP1-10 expression cassette. The position targeted by the gRNA within the pfs47 gene, regions of homology between the pfs47 locus and the repair plasmid, and binding sites for primer 1 and primer 2 used for PCR analysis are shown. (B) PCR analysis of parasites with GFP1-10 expression cassettes integrated into the pfs47 gene locus. PCR reactions were performed using genomic DNA from cloned parasites and primers 1 and 2 (taattgcatacacataaatatttgtgttgtac and ggagataaatgtaaggtaaatatacacaaac) and analysed using an ethidium bromide stained agarose gel. (TIF) [file ppat.1011281.s009.tif]
