# Supplementary material for: Formation of ER-lumenal intermediates during export of Plasmodium proteins containing transmembrane-like hydrophobic sequences
Source: PLoS Pathog. 2023 Mar 31;19(3):e1011281. doi: 10.1371/journal.ppat.1011281 (PMC10096305; doi:10.1371/journal.ppat.1011281)
Supplement: S1 Table — (DOCX) [file ppat.1011281.s010.docx]

**S1 Table**

Predicted transmembrane segments and ΔG values were determined using ΔG predictor (http://dgpred.cbr.su.se) [1-3].

| Protein | Predicted delta G | Predicted Transmembrane segment |
| --- | --- | --- |
| P02786\|TFR1_HUMAN Transferrin receptor protein 1 | -2.576 | ICYGTIAVIVFFLIGFMIGYLGY |
| P02724\|GLPA_HUMAN Glycophorin-A | -1.646 | ITLIIFGVMAGVIGTILLISYGI |
| P06028\|GLPB_HUMAN Glycophorin-B | -3.475 | APVVIILIILCVMAGIIGTILLI |
| MAHRP2 - PF3D7_1353200 | -3.679 | LMLIFLLLAFFLVVVYRLY |
| Pf332 - PF3D7_1149000 | -2.218 | TMVLVPGVLTVFLLTIIWVLVYK |
| SBP1 - PF3D7_0501300 | -3.599 | YLYVVIFLFFVINILLFINFYNL |
| REX2 - PF3D7_0936000 | -1.905 | CLPQVLFLYVIFLLLCTGIFM |
| MAHRP1 - PF3D7_1370300 | -3.994 | VFLLLFLFFGFVFCLLYHAFLY |
| RIFIN - PF3D7_1254800 | -5.428 | IILSAIAILVIVIIMVIIYLILR |
| STEVOR - PF3D7_0101800 | -6.073 | ITALVLLILAVLLIILYIWLYRR |
| PfEMP1 - PF3D7_0425800 | -2.177 | AMLFSTILWMVGIGFAAFTYFFL |
| PfEMP1 - PF3D7_1100200 | -1.859 | ALMSSTILWMVGIGFAALTYFLL |
| PfEMP1 - PF3D7_0425800 | -2.17 | AMLFSTILWMVGIGFAAFTYFFL |
| PfEMP1 - PF3D7_1373500 | -0.76 | TILQTTIPFGIALALGSIAFLFL |
| PfEMP1 - PF3D7_1300300 | -0.314 | ALMSSTIMWSVGIGFAAISYFLL |

1. Andersson A, Kudva R, Magoulopoulou A, Lejarre Q, Lara P, Xu P, et al. Membrane integration and topology of RIFIN and STEVOR proteins of the *Plasmodium falciparum* parasite. FEBS J. 2020;287(13):2744-62. Epub 2019/12/11. doi: 10.1111/febs.15171. PubMed PMID: 31821735.

2. Hessa T, Kim H, Bihlmaier K, Lundin C, Boekel J, Andersson H, et al. Recognition of transmembrane helices by the endoplasmic reticulum translocon. Nature. 2005;433(7024):377-81. Epub 2005/01/28. doi: 10.1038/nature03216. PubMed PMID: 15674282.

3. Hessa T, Meindl-Beinker NM, Bernsel A, Kim H, Sato Y, Lerch-Bader M, et al. Molecular code for transmembrane-helix recognition by the Sec61 translocon. Nature. 2007;450(7172):1026-30. Epub 2007/12/14. doi: 10.1038/nature06387. PubMed PMID: 18075582.
