# Supplementary material for: Formation of ER-lumenal intermediates during export of Plasmodium proteins containing transmembrane-like hydrophobic sequences
Source: PLoS Pathog. 2023 Mar 31;19(3):e1011281. doi: 10.1371/journal.ppat.1011281 (PMC10096305; doi:10.1371/journal.ppat.1011281)
Supplement: S1 Text — (DOCX) [file ppat.1011281.s011.docx]

**S1 Text**

**Characterisation of GFP_1-10_ protein expression in parasites**

To determine the fraction of cells within the population that express GFP_1-10_ proteins when expressed from a cassette integrated into the pfs47 gene, parasites expressing ER-lumenal GFP_1-10_ were transfected with a plasmid expressing ER-targeted mCherry with a C-terminal S11 tag (ER-lumenal mCherry comprises the N-terminal signal peptide derived from PF3D7_0827900, mCherry, a C-terminal S11 tag, and a STREP tag, followed by an SDEL sequence). When successfully co-expressed, the mCherry tagged protein with a C-terminal S11 tag should associate with the GFP_1-10_, resulting in parasites that are both red and green. Parasites were selected in the phase contrast view and imaged. As is typically found when proteins are expressed from plasmids, not all parasites expressed the ER-localised mCherry protein. Parasites that were fluorescent in the red channel were then scored based on whether they were also fluorescent in the green channel; in two independent experiments a total of 128 parasites were imaged and of these 100% of the parasites that expressed the red fluorescent protein were also fluorescent in the green channel (see table below). Similar results were obtained when cytoplasmic mCherry with and S11 tag was co-expressed with cytoplasmic GFP_1-10_.

| **Protein expressed from plasmid** | **GFP_1-10_ fragment expressed from integrated cassette** | **Number of parasites expressing mCherry protein** | **Number of parasites expressing mCherry protein also scored as fluorescent green** |
| --- | --- | --- | --- |
| Cytoplasmic mCherry:S11 | Cytoplasmic GFP_1-10_ | 128 | 128 |
| Cytoplasmic mCherry:S11 | ER lumenal GFP_1-10_ | 128 | 0 |
| ER-lumenal mCherry:S11 | Cytoplasmic GFP_1-10_ | 128 | 0 |
| ER-lumenal mCherry:S11 | ER lumenal GFP_1-10_ | 128 | 128 |
