# Supplementary material for: Formation of ER-lumenal intermediates during export of Plasmodium proteins containing transmembrane-like hydrophobic sequences
Source: PLoS Pathog. 2023 Mar 31;19(3):e1011281. doi: 10.1371/journal.ppat.1011281 (PMC10096305; doi:10.1371/journal.ppat.1011281)
Supplement: S2 Text — (DOCX) [file ppat.1011281.s012.docx]

**S2 Text**

**Mass spectrometry**

For the first two samples of REX3_RQLSE_:Pf332:_C-S11_:SDEL, proteins were first separated by SDS-PAGE. Bands from gels were cut into cubes and transferred to 1.5 mL Eppendorf tubes. Coomassie stain (Sigma-Aldrich) was removed with 3 washes of 50% ethanol in 50 mM ABC incubated for 20 minutes at 55˚C with 650 rpm shaking. Gel pieces were dehydrated with ethanol for 5 minutes with 650 rpm shaking. Proteins were reduced and alkylated with 10 mM TCEP and 40 mM CAA for 5 minutes at 70˚C. The gel pieces were then washed 3 times with 50% ethanol in 50 mM ABC for 20 minutes with 650 rpm shaking. Ethanol was added to dehydrate gel pieces, ethanol was removed before 2.5 ng/µg of trypsin in ABC was added per tube, and samples were left digesting overnight at 37˚C. Peptides were extracted from gel pieces with 25% ACN and sonicated for 10 minutes three times. Peptides were dried with Concentrator plus speed vac and resuspended in 10 µL of 3% ACN 0.1% FA and sent for MS analysis.

LC-MS/MS analysis was performed either on a Synapt G2-Si (Waters Corporation) or an Orbitrap Eclipse (Thermo Scientific). LC–MS/MS analysis was performed using an Acquity M series and a Synapt G2-Si (Waters Corporation). 5 µL of sample with 1 *μ*g of peptides, was loaded on a reversed-phase Symmetry C18 trap column (180 μm internal diameter, 20 mm length, 5 μm particle size, Waters Corporation) at a flow rate of 8 μL/min for 3 min in 0.1% formic acid, for 3 min at 8 *μ*L/min. Mobile phase A was of 0.1% formic acid and mobile phase B was ACN containing 0.1% formic acid. Peptides were then separated using a linear gradient (0.3 μL/min, 35˚C; 97−60% buffer A over 90 min) using a BEH130 C18 nanocolumn (75 μm internal diameter, 250 mm length, 1.7 μm particle size, Waters Corporation). The TOF analyser was externally calibrated from m/z 175.11 to 1285.54 using [Glu1]-fibrinopeptide B at 500 fmol/μL. To avoid cross-contamination between samples, a minimum of two washes of 30 min each was run between samples.

Data-Dependent Acquisition. Samples were analysed using a Waters Synapt G2-Si quadrupole time-of-flight mass spectrometer tuned to a resolution of 20,000 (fwhm). Accurate mass measurements were made using DDA. The top 10 most intense precursors with charge states between +3 and +7 were selected over a mass range of 50−3000 Da with a scan time of 0.15 s and an interscan delay of 0.05 s. MS2 spectra were acquired using collision energy ramps low mass 10-20 eV high mass 30-60 eV. Dynamic exclusion was used with a 30 s window to prevent repeated selection of peptides.

Raw data files (Waters) were searched using Progenesis using the following parameters: trypsin as the digestion enzyme, with up to 3 missed cleavages, and a parent ion mass tolerance of 10 ppm. Oxidation of Met and Acetylation of the N-terminal were set as variable modifications and Carbamidomethyl of Cys as a fixed modification for all searches.

For analysis of the third sample of REX3_RQLSE_:Pf332:_C-S11_:SDEL, proteins were eluted from Streptactin beads and digested with trypsin in solution. The mixture of tryptic peptides was analysed using an Ultimate3000 high-performance liquid chromatography system coupled online to an Eclipse mass spectrometer (Thermo Fisher Scientific). Buffer A consisted of water acidified with 0.1% formic acid, while buffer B was 80% acetonitrile and 20% water with 0.1% formic acid. The peptides were first trapped for 1 min at 30 μl/min with 100% buffer A on a trap (0.3 mm by 5 mm with PepMap C18, 5 μm, 100 Å; Thermo Fisher Scientific); after trapping, the peptides were separated by a 50-cm analytical column (Acclaim PepMap, 3 μm; Thermo Fisher Scientific). The gradient was 7 to 35% B in 44 min at 300 nl/min. Buffer B was then raised to 55% in 2 min and increased to 95% for the cleaning step. Peptides were ionized using a spray voltage of 2.1 kV and a capillary heated at 280°C.

The mass spectrometer was set to acquire full-scan MS spectra (350 to 1400 mass/charge ratio) for a maximum injection time set to Auto at a mass resolution of 60,000 and an automated gain control (AGC) target value of 100%. For a second the most intense precursor ions were selected for MS/MS. HCD fragmentation was performed in the HCD cell, with the readout in the Orbitrap mass analyser at a resolution of 15,000 (isolation window of 2 Th) and an AGC target value of 200% with a maximum injection time set to Auto and a normalized collision energy of 30%. Raw files were analysed by MaxQuant v2.1.4 software using the integrated Andromeda search engine. MaxQuant was used with the standard parameters with only the addition of deamidation (N) as variable modification. Data analysis was then carried out with Perseus v2.05.

The mass spectrometry proteomics data have been deposited to the ProteomeXchange Consortium via the PRIDE (Perez-Riverol et al., 2019) partner repository with the dataset identifier PXD036904.
